# Unlocking the Role of C Doping in a RuO_2_ Matrix in CO_2_ Methanation from a Combined Theoretical and Experimental Approach

**DOI:** 10.1021/acs.jpcc.5c01277

**Published:** 2025-03-12

**Authors:** Alvaro Royo de Larios, Carmen Tébar-Soler, Daviel Gómez, Patricia Concepción, Mercedes Boronat, Avelino Corma

**Affiliations:** Instituto de Tecnología Química, Universitat Politècnica de València − Consejo Superior de Investigaciones Científicas, Avenida de los Naranjos s/n, València 46022, Spain

## Abstract

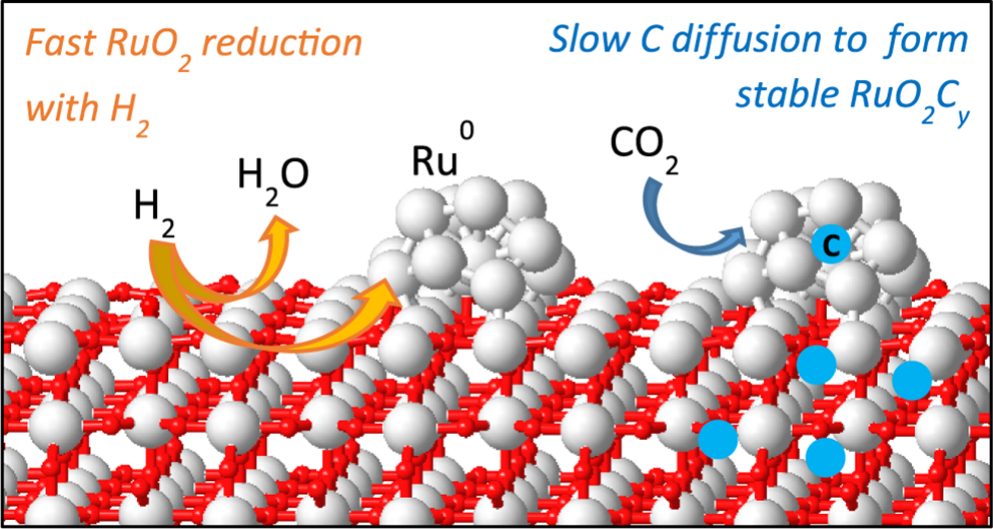

A new type of ruthenium-based catalyst, labeled RuO_*x*_C_*y*_@C, consisting of a combination of metallic ruthenium (Ru^0^), ruthenium oxide (RuO_2_), and a ruthenium oxycarbonate phase (RuO_2_C_*y*_) formed by interstitial carbon doped into RuO_2_, has been recently reported for low-temperature CO_2_ methanation. Its catalytic activity and long-term stability depend on two competing processes that take place under reaction conditions: RuO_2_ reduction with H_2_ to form inactive Ru^0^ nanoparticles, and C diffusion into RuO_2_ to form the active oxycarbonate phase. A combination of experimental and computational techniques is applied in this work to investigate the relative rate of both processes in order to identify possible modifications in the catalyst composition that might improve the overall catalytic performance.

## 1 Introduction

With the continuous transition from conventional to renewable energy sources, adequate energy storage technologies are crucial to mitigate the intermittent behavior of renewable energy. One promising storage technology is the Power to Gas system (PtG), converting a mixture of H_2_ and CO_2_ into synthetic natural gas (i.e., methane).^1−3^ Using natural gas over H_2_ has the advantage of an existing natural gas infrastructure and available end-use technologies. Although PtG is a well-established technology, lowering the cost of methane production remains a challenge, which involves decreasing both the cost of producing H_2_ via electrolysis and the cost of the methanation process.^4,5^ Regarding the latter, current processes operate at high temperatures between 250 and 400 °C, with significant drawbacks regarding catalyst stability and energy efficiency. In this direction, we recently discovered a new type of ruthenium-based catalyst (labeled as RuO_*x*_C_*y*_@C)^6^ that operates at low temperatures (160–180 °C) with competitive methane yields compared to state-of-the-art Ru and Ni catalysts.^7^ The catalyst is composed of three coexisting phases: metallic Ru^0^, ruthenium oxide RuO_2_, and a novel oxy-carbonate ruthenium phase (RuO_2_C_*y*_) where C is doped at interstitial positions of the RuO_2_ crystal lattice. This catalyst is stable under continuous reaction conditions for at least 10 days of operation, operating at 180 °C, 20 bar, and a space velocity of 24000 h^–1^. Spectroscopic characterization using X-ray photoelectron spectroscopy (XPS) and X-ray absorption spectroscopy (XAS) has shown a key role of C in stabilizing ruthenium atoms in a low oxidation state (i.e., + 2.5), similar to that observed on homogeneous catalysts,^8^ which has been reported to activate CO_2_ under mild reaction conditions. The reported catalyst is prepared by a hydrothermal synthesis approach, while preliminary studies have shown that the active RuO_2_Cy phase can also be generated *in situ* under reaction conditions starting from RuO_2_ as a precatalyst. The catalytic activity and stability can be easily adjusted by modifying the reaction conditions,^9^ such as reaction temperature (140–180 °C), pressure (1–20 bar), and space velocity (6000–120000 h^–1^). This is due to two parallel processes that compete under reaction conditions, i.e., carbon diffusion leading to the stabilization of the oxycarbonate phase and RuO_2_ reduction into metallic Ru^0^. The extent of both processes affects the catalyst’s final state and consequently its reactivity. Therefore, the current study aims to analyze each of these processes by combining DFT analysis with experimental studies, paving the way for the design of more stable catalysts.

## 2 Experimental Section

### 2.1 Synthesis of the RuO*_x_*C*_y_*@C Sample

RuO_*x*_C_*y*_@C catalyst was prepared by a hydrothermal synthesis method reported in ref. (6) In detail, 120 mg of glucose and 100 mg of RuO_2_ (Aldrich, 39 nm) were dissolved in 7 mL of Milli-Q water. Then, the mixture was loaded into a Teflon-coated stainless-steel autoclave of 15 mL. The autoclave was introduced into an oven under static conditions at 175 °C for 24 h. After cooling down to room temperature over 2 h, the content of the autoclave was filtered under vacuum conditions. The solid was washed, first with distilled water until no foam was observed and, later, one time with acetone. Finally, it was dried in an oven at 60 °C for 12 h.

### 2.2 Catalyst Characterization

X-ray powder diffraction (XRD) was recorded with a Philips X́Pert diffractometer using monochromatic Cu Kα radiation (λ = 0.15406 nm).

X-ray photoelectron spectra (XPS) were collected using a SPECS spectrometer equipped with an MCD-9 detector and a non-monochromatic AlKα (1486.6 eV) X-ray source. Spectra were recorded using an analyzer pass energy of 10 eV, an X-ray power of 100 W, and an operating pressure of 10^–9^ mbar. Data processing was performed using the CASA software and a Shirley line as the background. For ruthenium core levels, a tail-dampened Lorentzian asymmetric line shape (LF (α, β, ω, *m*)) has been used, where α and β define the spread of the tail on each side of the Lorentzian component, ω determines the tail-dampening parameter, and *m* the width of the Gaussian used to convolute the Lorentzian curve. In particular, values of LF (0.8, 1.25, 500, 180) for Ru^0^ and LF (0.6, 1.6, 50, 150) for RuO_2_ have been used.^6^ For C 1s, a Gaussian (70%)—Lorentzian (30%) curve defined as GL (30) has been used. Spectra were calibrated with respect to C 1s settled at 284.5 eV.

### 2.3 Reducibility Experiments

The reducibility of the catalyst was analyzed in a stainless-steel fixed-bed reactor with an inner diameter of 6.5 mm and a length of 133 mm. Two types of experiments were performed: one isothermal at 100 °C, stopping the reduction process at 1 and 30 min and analyzing the final catalyst by XRD, and another under temperature-programmed conditions, increasing the reduction temperature from 25 to 350 °C by following the evolution of water by online mass spectrometry (MS). In the first case, 180 and 90 mg of RuO_2_ and RuO_*x*_C_*y*_@C, respectively (particle size 400–600 μm), were diluted in 1000 mg of SiC (600–800 μm) and mounted inside the reactor without activation. The experiment took place at 1 bar and 100 °C, with a gas mixture of 10 vol. % H_2_ and 90 vol. % N_2_ (50 mL/min). In the second case, 50 mg of catalyst was exposed to a flow of 15 mL/min of 33% H_2_/He mixture and then the temperature was increased from 25 to 350 °C. The apparent activation energy of the reduction process was determined by analyzing the temperature at the maximum reduction rate (*d*α/*d*t = Max → *T*_Max_) or the temperature at a specific conversion value (α = 10% → *T*_α=10%_) according to the method used for the calculation at variable heating rates (4, 8, 10, and 12 °C/min).^10^ The procedure and mathematical expressions of each method are summarized in Section 2 and Table S1.

### 2.4 Catalytic Experiments

CO_2_ hydrogenation was performed in a stainless-steel fixed-bed reactor with an inner diameter of 11 mm and a length of 240 mm. Typically, 260 mg of catalyst (particle size 400–600 μm) was diluted in 6300 mg of SiC (600–800 μm) and mounted inside the reactor without activation. The reaction took place at 20 bar and 180 °C. The reaction was carried out at a GHSV of 24000 h^–1^. The inlet gas mixture was 23.8 vol. % CO_2_, 71.3 vol. % H_2_, and 5 vol. % N_2_ (1:3 CO_2_:H_2_). Direct analysis of the reaction products was done by online gas chromatography using SCION-456-GC equipment with TCD (MS-13X column) and FID (BR-Q Plot column) detectors. For time-resolved studies, a microreactor of 6.5 mm diameter and 133 mm length was used. In this type of experiment, 90 mg of catalyst was used, diluted in 1000 mg SiC, and the reaction temperature was increased from 25 to 160 °C, with a rate of 10 °C/min, in a CO_2_/H_2_ flow (1:3) operating at 10 bar. Once the final temperature was achieved, the reaction was stopped after 1 and 30 min, followed by cooling to room temperature in N_2_ flow. Direct analysis of the reaction products was done by online gas chromatography using Agilent 8860-GC equipment with TCD (HP-Plot/Q and HP-Molsieve columns) and FID (HP-Plot/U column) detectors. The final catalyst was analyzed by XRD.

### 2.5 Computational Details

#### 2.5.1 Models

Different models were used in this work to investigate the bulk and surface properties of ruthenium oxide (RuO_2_), ruthenium oxycarbonate (RuO_2_C_*y*_) and metallic ruthenium (Ru^0^).

**Figure 1:**
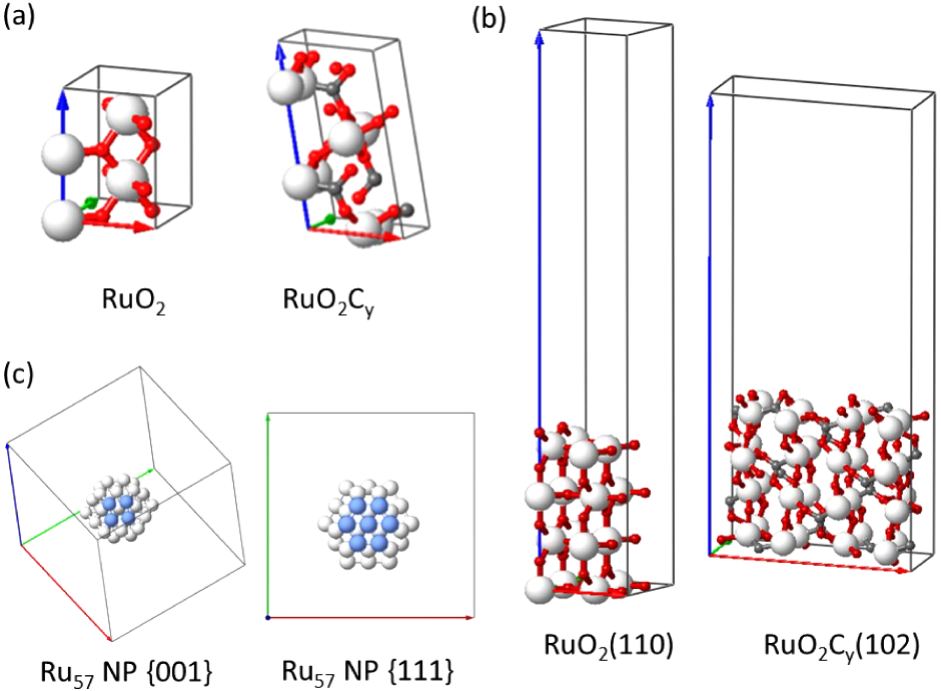
Models used in the theoretical study. (a) Bulk RuO_2_ and RuO_2_C_*y*_ unit cells. (b) Slab models of the RuO_2_(110) and RuO_2_C_*y*_(102) surfaces. (c) Ru_57_ nanoparticles with the exposed {001} and {111} planes highlighted in blue. Ru, O, and C atoms are depicted as white, red, and gray balls, respectively.

The tetragonal unit cell of bulk RuO_2_ employed in this work contains 4 ruthenium and 8 oxygen atoms, and the optimized lattice parameters are *a* = *b* = 4.478 Å, *c* = 6.181 Å, and α = γ = β = 90° (Figure 1a). The bulk crystallographic structure of RuO_2_C_*y*_ is monoclinic and was taken from ref. (6). The unit cell contains 8 ruthenium, 16 oxygen, and 4 carbon atoms, in accordance with the experimental empirical formula RuO_2_C_0.41_, and the optimized lattice parameters are *a* = 5.199 Å, *b* = 5.761 Å, *c* = 10.204 Å, α = 89.710°, β = 104.002°, and γ = 90.152° (Figure 1a). These two models were used to investigate the reducibility of the bulk materials via successive removal of the O atoms, generating an increasing amount of O vacancy defects. In these calculations, the positions of all atoms in the system and the unit cell parameters were allowed to relax without constraints, and the Brillouin zone was sampled with a gamma-centered 7 × 7 × 3 k-points mesh.

Following the experimental information, the most stable (110) surface of RuO_2_ and the (102) surface of the RuO_2_C_*y*_ phase, which corresponds to the (110) surface of RuO_2_, were cleaved from the bulk to serve as models for the reactivity studies (Figure 1b). The RuO_2_(110) surface model contains 16 ruthenium and 32 oxygen atoms in four layers, and the unit cell parameters are *a* = 6.402 Å, *b* = 6.285 Å, and *c* = 38.416 Å. The RuO_2_C_*y*_(102) surface model contains 32 ruthenium, 64 oxygen, and 16 carbon atoms in four layers, and the unit cell parameters are *a* = 16.235 Å, *b* = 5.761 Å, and *c* = 36.894 Å. In both slab models, 1/3 of the unit cell volume is occupied by Ru, O, and C atoms, and the other 2/3 of the unit cell volume is empty to avoid interactions between periodically repeated slabs. During geometry optimizations, the positions of the atoms in the two topmost layers were allowed to relax, and those in the two bottom layers were kept fixed at their bulk positions to avoid spurious deformations. In these calculations, the Brillouin zone was sampled with a 4 × 4 × 1 k-points mesh for RuO_2_(110) and with a 2 × 4 × 1 k-points mesh for the larger RuO_2_C_*y*_(102) model.

Metallic Ru^0^ was simulated by means of a hcp-based nanoparticle of 57 atoms (Ru_57_) of ∼1.1 nm, exposing {111} and {001} planes, that was placed in a 25 × 25 × 25 Å^3^ cubic box (Figure 1c), following previous work by Comas-Vives et al.^11^ The Brillouin zone was sampled with a gamma-centered 1 × 1 × 1 k-points mesh, and all atoms in the system were allowed to relax during geometry optimizations.

#### 2.5.2 Methods

All calculations were performed using periodic density functional theory (DFT) and the Vienna Ab initio Simulation Package (VASP).^12,13^ We applied the GGA-type (Generalized Gradient Approximation) functional of Perdew, Burke, and Ernzerhof (PBE).^14^ The valence density was expanded in a plane wave basis set with a kinetic energy cutoff of 400 eV for the Ru_57_ metallic nanoparticle and 500 eV for every RuO_2_ and RuO_2_C_*y*_ model. The interaction between core electrons and valence electron density was taken into account by means of the projector augmented wave formalism (PAW).^15,16^ The electrons included in the valence space were 5s^1^4d^7^ for Ru, 2s^2^2p^4^ for O, 1s^1^ for H, and 2s^2^2p^2^ for C. The convergence criteria were 10^–6^ eV for the SCF energy and 0.01 eV/Å in the maximal force per atom. The Gaussian smearing method was employed to determine electron occupancies with a smearing parameter of σ = 0.01.

All calculations are spin-polarized. Atomic charges were obtained through the Bader charge analysis.^17−19^ Transition state structures were obtained using the DIMER^20,21^ method as implemented in VASP. All minima and transition states were characterized by means of frequency calculations, calculated numerically through central differences of analytically derived forces.

Free energies were obtained from the partition functions of the system, calculated from the vibrational energies obtained in the harmonic approximation using VASP. For the solid components, only vibrational contributions are considered, whereas for gas-phase species, rotational and translational contributions are also included. The free energy values reported throughout the manuscript were calculated at 160 °C (433.15 K), which is the temperature at which the CO_2_ methanation was carried out in the time-resolved studies.

Oxygen vacancy formation energies (E_v_) were calculated as
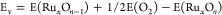

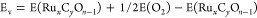


where E(Ru_*x*_O_*n*_) and E(Ru_*x*_C_*y*_O_*n*–1_) are the total energies of the RuO_2_ and RuO_2_C_*y*_ models, E(Ru_*x*_O_*n–*1_) and E(Ru_*x*_C_*y*_O_*n–*1_) are the total energies of the same systems after the removal of one oxygen atom, and E(O_2_) is the total energy of molecular O_2_ in its triplet state.

## 3 Results and Discussion

### 3.1 Stability of RuO_2_ and RuO_*x*_C_*y*_@C Catalysts under Reaction Conditions

**Figure 2:**
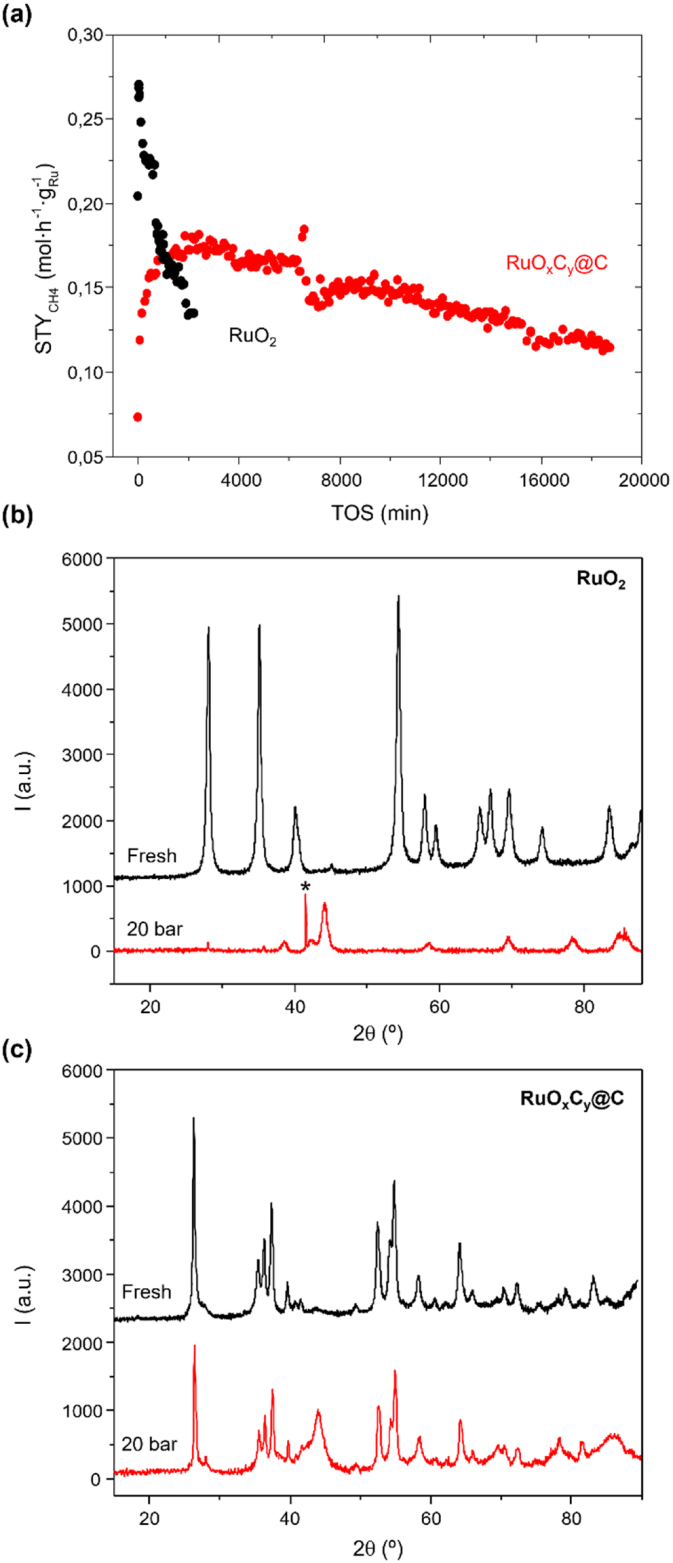
(a) Space time yield of methane (STY) in mol h^–1^ g_Ru_^–1^ over RuO_2_ (in black) and RuO*_x_*C*_y_*@C (in red) samples. Reaction conditions: 20 bar, 180 °C, 1:3 CO_2_:H_2_, and 24000 h^–1^. Deactivation rates for both catalysts are given in Figure S1. (b) XRD pattern of fresh RuO_2_ (in black) and after being exposed to the reaction conditions (in red). (c) XRD pattern of fresh RuO*_x_*C*_y_*@C (in black) and after exposure to reaction conditions (in red). The asterisk indicates peaks due to SiC, which has been used as diluting agent in the catalytic studies.

The RuO_*x*_C_*y*_@C catalyst was prepared by a hydrothermal synthesis method reported in ref. (6). The final material is composed of the coexistence of three phases, RuO_2_C_*y*_/RuO_2_/Ru^0^ in a 60/30/10 mol ratio, based on Rietveld XRD analysis and MCR-XAS studies. The catalytic activity in the CO_2_ hydrogenation to methane at 20 bar, CO_2_:H_2_ 1:3, and GHSV 24000 h^–1^ is shown in Figure 2a.^6^

As observed, starting directly from RuO_2_ (Aldrich, 39 nm), the activity of the catalyst increases in the first 60 min of reaction and then decreases with increasing reaction time. In contrast, the RuO_*x*_C_*y*_@C catalyst behaves more stably over 11 days of operation days, with a slight deactivation (more details in Section 1 of the Supporting Information).

The XRD pattern of the RuO_2_ catalyst, after having been exposed to reaction conditions, shows an almost complete transformation of RuO_2_ to inactive Ru^0^ (Figure 2b) whereas, in the case of the RuO_*x*_C_*y*_@C catalyst, some Ru^0^ in coexistence with the active RuO_2_C_*y*_ phase is observed (Figure 2c). In view of these results, the different catalytic trend observed in Figure 2a can be explained by the fact that the oxycarbonate ruthenium phase in the RuO_*x*_C_*y*_@C catalyst remains stable under reaction conditions, whereas when starting from RuO_2_, metallic Ru^0^ prevails under steady-state reaction conditions. The reason behind the different behavior of the two materials has not yet been examined but can be explained if two hypotheses are accepted: first, that the reduction of ruthenium oxide is a fast process and, second, that the C atoms in the RuO_2_C_*y*_ phase contribute to slowing down the kinetics of reduction. These hypotheses have been validated in this study from an experimental and theoretical perspective. Hence, from an experimental point of view, two types of experiments were conducted, where both RuO_2_ and RuO_*x*_C_*y*_@C catalysts were exposed to controlled reaction conditions, i.e., a reactant flow (CO_2_/H_2_ = 1/3) and a H_2_ flow (10% in N_2_) with stopping the reaction at 1 and 30 min, and analyzing the initial and final state of the catalysts by XRD.

**Figure 3:**
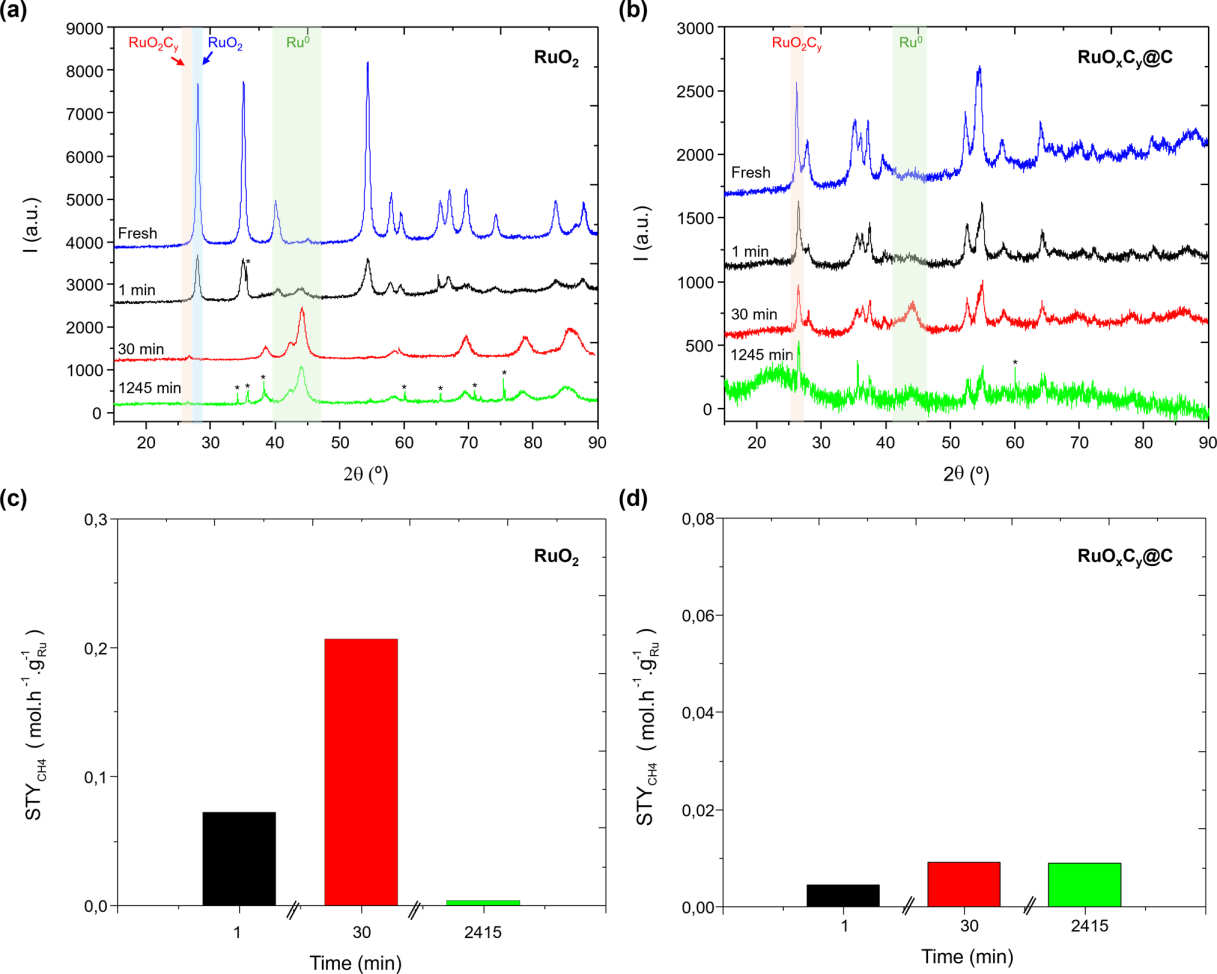
(a, b) XRD pattern of RuO_2_ (a) and RuO*_x_*C*_y_*@C (b) samples: fresh (in blue) and after having been exposed to reaction conditions (CO_2_/H_2_ 1:3 at 10 bar, 160 °C and 60000 h^–1^) for 1 min (in black), 30 min (in red), and 1245 min (in green). (c, d) STY_CH4_ at different times of reaction for RuO_2_ (c) and RuO*_x_*C*_y_*@C (d) samples. The full catalytic data with the time of stream are given in Figure S2. The asterisk in (a, b) indicates peaks due to SiC, which has been used as a diluting agent in the catalytic studies. The red, blue, and green zones indicate the main peak of the RuO_2_C_*y*_, RuO_2_, and Ru^0^ phases, respectively.

Figure 3 shows the XRD pattern and associated methane production determined by GC analysis of the samples after having been exposed to reaction conditions (i.e., CO_2_/H_2_ flow) at controlled reaction times. In order to slow down the kinetics of the reaction, experiments were done at lower temperature and pressure (i.e., 160 °C and 10 bar). When starting from RuO_2_ (Figure 3a), after 1 min, RuO_2_ (main peak at 28°) coexists with metallic Ru^0^ (main peak at 44°), whereas after 30 min of reaction, the peak at ∼26° associated with oxycarbonate, which had not been observed before, starts to be visible, although at a low intensity. The presence of this peak confirms the *in situ* formation of the ruthenium oxycarbonate phase when starting from RuO_2_ as a precatalyst, which is a slow process compared to the reduction of RuO_2_ to metallic Ru^0^. This peak disappears at increasing reaction time (∼1245 min) with the detection of exclusively metallic Ru^0^ in the XRD pattern. The methane production at each time is given in Figure 3c, showing an increase in the catalytic activity after 30 min, followed by a decrease after 1245 min, confirming RuO_2_C_*y*_ as the active phase. Notice that this behavior is not observed when operating at 180 °C and 20 bar (Figure 2) due to a fast reduction of the catalyst concomitant with its loss of activity. In contrast, starting from RuO_*x*_C_*y*_@C, the peaks of RuO_2_C_*y*_ remain stable even after 1245 min of reaction, with metallic Ru^0^ formed but to a lesser (Figure 3b), supporting the higher catalytic stability of this sample (Figure 3d). A more detailed analysis of the catalyst surface can be obtained from XPS studies (Figure S3). Deconvolution of the C 1s+Ru 3d peak in the fresh RuO_2_ sample shows several components at 281.2, 282.7, and 283.4 eV associated with oxidized ruthenium species,^22−25^ while in the spent sample after 30 min of reaction, two new components are observed at 279.6 and 280.3 eV, associated with ruthenium metal (3.4 atomic %) and the RuO_2_C_*y*_ phase (12.3 atomic %), respectively.^26^ These data clearly indicate the *in situ* formation of the active RuO_2_C_*y*_ phase under reaction conditions, which is hardly observed from the XRD data due to their lower surface sensitivity (see Table 1 and Figure S3). In the RuO_*x*_C_*y*_@C sample, ruthenium oxycarbonate (RuO_2_C_*y*_) is observed in the fresh sample at 3.3 atomic %, together with ruthenium oxide (281.5 eV). During the reaction, RuO_2_C_*y*_ increases to 8.3 atomic % after 1 min and then decreases to 2.7 atomic % after 30 min of reaction. Notice that due to the different surface sensitivities of the XPS and XRD techniques, the concentration of RuO_2_C_*y*_ determined by XPS analysis is different from that obtained from the XRD pattern. While RuO_2_C_*y*_ is a bulk phase in the RuO_*x*_C_*y*_@C sample and therefore easily detected by XRD, it is formed in situ when starting from RuO_2_. This formation takes place preferentially on the surface, and therefore it is hard to detect by XRD. Furthermore, XPS data show an increase in the Ru/C atomic ratio in the RuO_*x*_C_*y*_@C sample under reaction conditions, due to the removal of some amorphous carbon coming from the hydrothermal synthesis.^6^

**Table 1:** Surface Composition Determined by XPS by Deconvolution of the C 1s and Ru 3d Core Levels on Fresh and Spent RuO_2_ and RuO_*x*_C_*y*_@C Samples^a^

	**C 1s**			**Ru 3d**5/2					
Sample	CH	COH	RCOOH	Ru^0^	RuO_*x*_C_*y*_	Ru^IV^	s.Ru^IV^	Ru^VI^	Ru/C
**RuO**_*x*_**C**_*y*_**@C Fresh**	284.5	287.4	288.7		280.3	281.5			0.06
	(53.9)	(31.7)	(8.8)		(3.3)	(2.2)			
**RuO**_*x*_**C**_*y*_**@C 1 min**	284.5	286.7	288.4	279.7	280.4	281.9			0.11
	(72.7)	(10.8)	(6.4)	(0.2)	(8.3)	(1.5)			
**RuO**_*x*_**C**_*y*_**@C 30 min**	284.5	286.7		279.0	280.3				0.20
	(57.7)	(25.5)		(14.0)	(2.7)				
**RuO**_**2**_**Fresh**	284.5	285.8				281.2	282.7	283.5	0.25
	(19.9)	(59.8)				(14.6)	(4.4)	(1.2)	
**RuO**_**2**_**30 min**	284.7	285.4	288.0	279.6	280.3		282.1		0.26
	(19.6)	(48.1)	(11.3)	(3.4)	(12.3)		(5.0)		

a The numbers in parentheses represent the surface atomic percentage of the components.

### 3.2 Reducibility of RuO_2_ and RuO_*x*_C_*y*_@C Catalysts in H_2_

**Figure 4:**
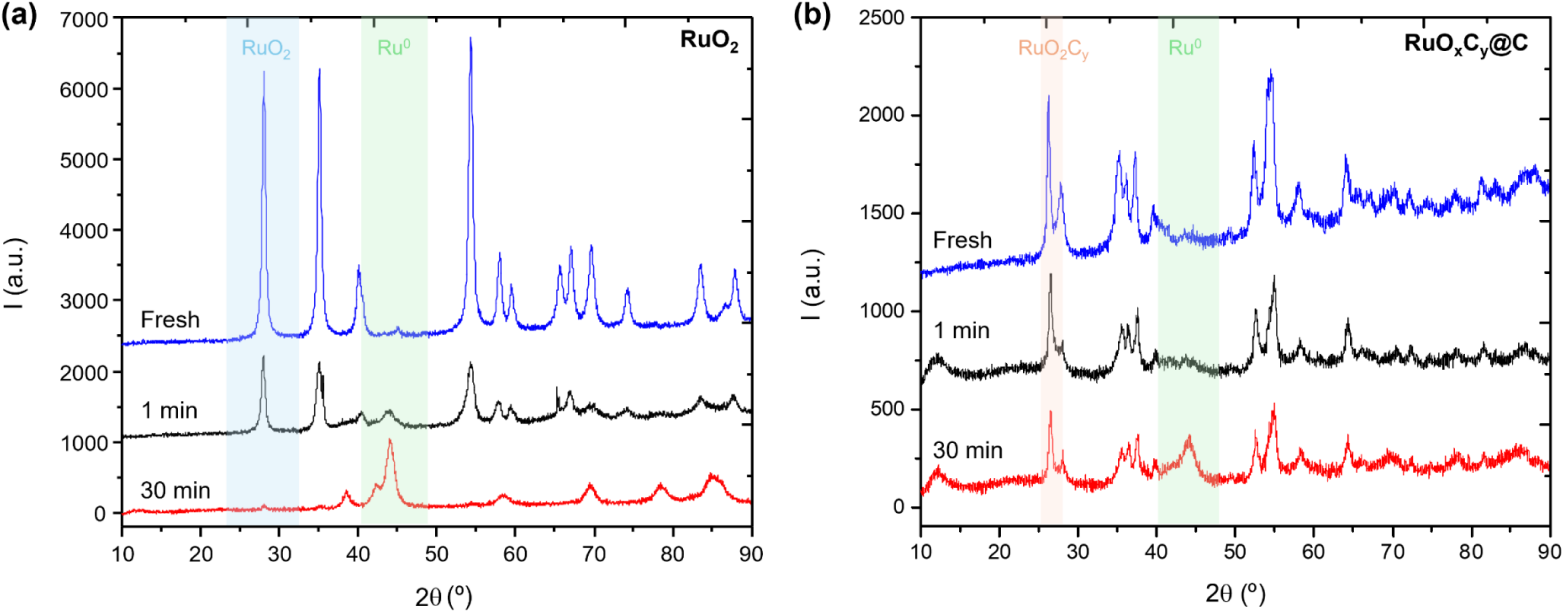
XRD pattern of (a) RuO_2_ and (b) RuO*_x_*C*_y_*@C samples: fresh (in blue) and after having been exposed to 10% H_2_ flow (in N_2_) at 100 °C and 1 bar, for 1 min (in black) and 30 min (in red). The blue, green, and orange zones indicate one of the main peaks of the RuO_2_, Ru^0^, and RuO_2_C_*y*_ phases, respectively.

This initial experiment indicates a different reducibility of the starting precursors, which is confirmed in a second experiment conducted in the presence of only H_2_ flow (10% in N_2_). In this case, the temperature was set to 100 °C, based on the TPR-H_2_ profile of Figure S4, and the reaction was stopped at 1 and 30 min. Under these conditions, and confirming the previous experiment, a fast and almost complete reduction to metallic Ru^0^ is observed after 30 min in the RuO_2_ sample (Figure 4a), while it is partially inhibited in the RuO_*x*_C_*y*_@C sample (Figure 4b). Altogether, these two experiments indicate that C doping slows down the reduction process, enabling the stabilization of the active oxycarbonate phase.

### 3.3 Kinetics of RuO_2_ and RuO_*x*_C_*y*_@C Catalyst Reduction with H_2_

**Figure 5:**
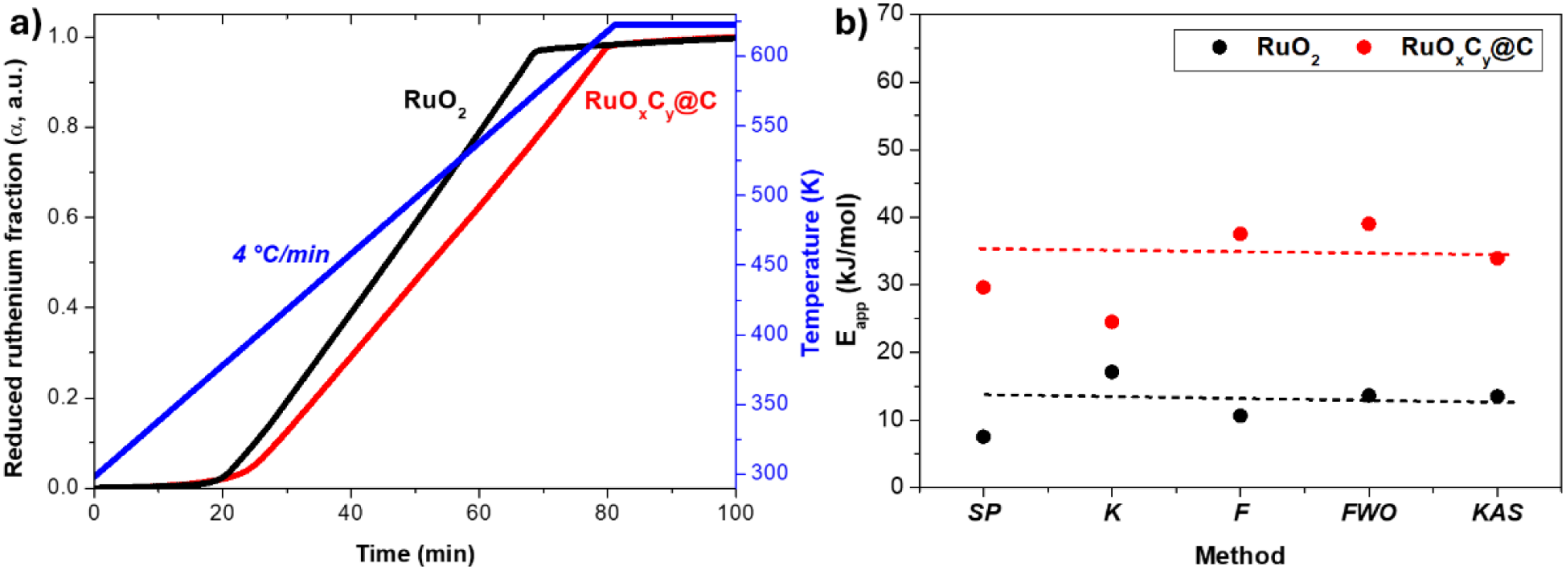
(a) Degree of reduction of RuO_2_ (in black) and RuO*_x_*C*_y_*@C (in red) samples under hydrogen atmosphere as a function of time and temperature, at a heating rate of 4 °C/min. (b) Apparent activation energy for the reduction of RuO_2_ (in black) and RuO*_x_*C*_y_*@C (in red) under hydrogen atmosphere as a function of the method used to calculate (SP: Stationary point method, K: Kissinger method, F: Friedman method, FWO: Flynn–Wall–Ozawa method and KAS: Kissinger–Akahira–Sunose method). Details of the methods are provided in the Supporting Information.

Next, the kinetics of reduction of the two materials were calculated using a catalytic reactor connected online to a MS and following the evolution of H_2_O (*m*/*z* 18) at increasing temperatures (details in the Experimental Section). The reduced fraction of ruthenium (α) as a function of temperature is displayed in Figures 5a and S5, and the apparent activation energy of the reduction process is given in Figure 5b. Following the methodology presented in ref. (10) and detailed in Section 3 of the Supporting Information, apparent activation energies of approximately 12.6 kJ/mol vs 36.8 kJ/mol were obtained for the RuO_2_ and RuO_*x*_C_*y*_@C samples, respectively (see Figure S6 and Table S2), in line with their different reducibility.

### 3.4 DFT Study of RuO_2_ and RuO_*x*_C_*y*_ Intrinsic Reducibility: Bulk and Exposed Surfaces

**Figure 6:**
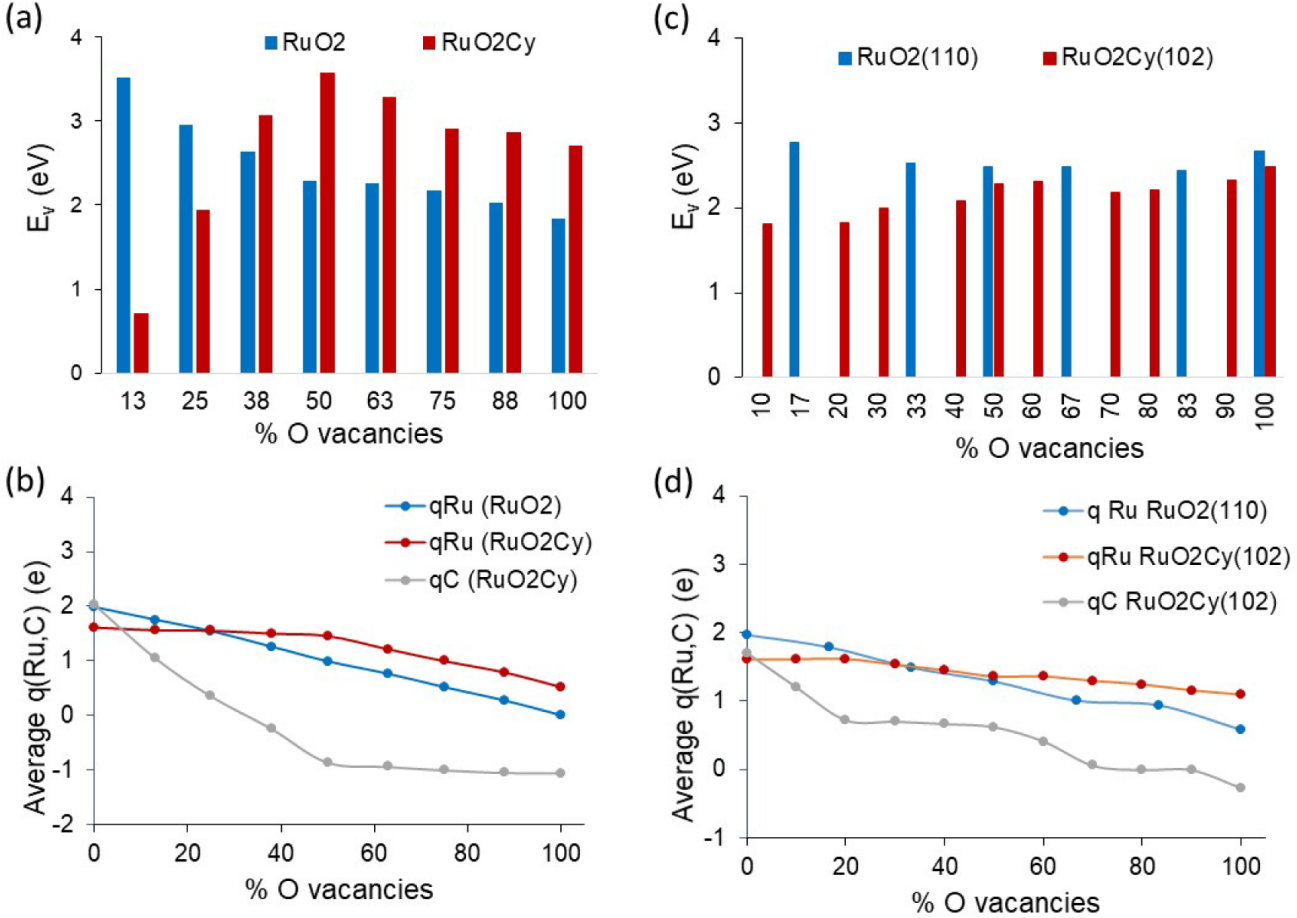
(a) Vacancy formation energies (E_v_) and (b) average charge on Ru (qRu) and C atoms (qC) with increasing percentage of oxygen vacancies in bulk RuO_2_ and RuO_2_C_*y*_ models. (c) Vacancy formation energies (E_v_) and (d) average charge on Ru (qRu) and C atoms (qC) with increasing percentage of oxygen vacancies in RuO_2_(110) and RuO_2_C_*y*_(102) surface models.

In the first step, the intrinsic reducibility of the bulk RuO_2_ and RuO_2_C_*y*_ phases was evaluated by calculating the thermodynamics of the successive removal of all O atoms in the unit cell, leading to the final formation of bulk Ru and bulk RuC_2_, respectively. Every O atom removed from the system leaves two electrons in the vacancy defect that are accepted by the Ru atoms, which results in a lower oxidation state of the metal. Since not all the O atoms in the unit cell are equivalent, individual vacancy formation energies (E_v_) were calculated for each distinct O atom at each composition, and the most stable system was taken as the initial structure to remove the subsequent O atom. The plots in Figure 6 show relevant differences between the RuO_2_ and RuO_2_C_*y*_ phases. The energy necessary to remove O atoms from bulk RuO_2_ decreases continuously as the material is reduced (blue bars in Figure 6a), in parallel with a constant decrease in the average charge on Ru atoms, from +2 in the initial RuO_2_ state, corresponding to the Ru(IV) oxidation state, to 0 in the final metallic Ru^0^ state (blue line in Figure 6b). In contrast, the removal of the first O atoms from bulk RuO_2_C_*y*_ requires little energy and becomes more difficult as the material loses oxygen, reaching a maximum at 50% O vacancies (red bars in Figure 6a). During this process, the net charge on Ru atoms remains constant at around +1.6, and it is the C atoms that accept the electrons left in the solid by the leaving O. As a consequence, the average atomic charge on the C atoms decreases from +2 in bulk RuO_2_C_*y*_ to −1 at 50% O vacancies (gray line in Figure 6b). After this reduction of the C atoms to an anionic state, subsequent removal of O in RuO_2_C_*y*_ leads to a smooth partial reduction of Ru atoms, which ends with an average charge qRu of +0.5 (red line in Figure 6b). These results confirm that the presence of interstitial C atoms in the RuO_2_C_*y*_ phase stabilizes Ru atoms in a low oxidation state, preventing their full reduction to metallic Ru^0^.

Similar but less pronounced trends were found when studying the thermodynamics of the reduction of the surfaces preferentially exposed by each material, namely, the RuO_2_(110) and the RuO_2_C_*y*_(102) facets. Notice that while there are only six distinct O atoms on the RuO_2_(110) facet, the presence of C in RuO_2_C_*y*_(102) reduces the symmetry, and as a result, there are ten distinct O atoms in the surface layer of the unit cell (see Figure S7). Each of them was individually removed, generating an O vacancy, and the most stable system was taken as the initial structure to remove the subsequent O atom, as done before to study bulk reducibility. The bar plots in Figure 6c indicate that it is slightly more favorable energetically to remove O atoms from the RuO_2_C_*y*_(102) surface than from the RuO_2_(110) facet. Figure 6d shows that while the net charge on Ru atoms in RuO_2_(110) decreases continuously from +2 to +0.5 as surface O are removed, the C atoms in RuO_2_C_*y*_(102) surface accept the additional electrons, becoming anionic and keeping the charge on Ru relatively stable above +1.

### 3.5 Mechanism of H_2_ Reaction with RuO_2_(110), RuO_2_C_*y*_(102), and Ru_57_ NP Catalyst Models

**Figure 7:**
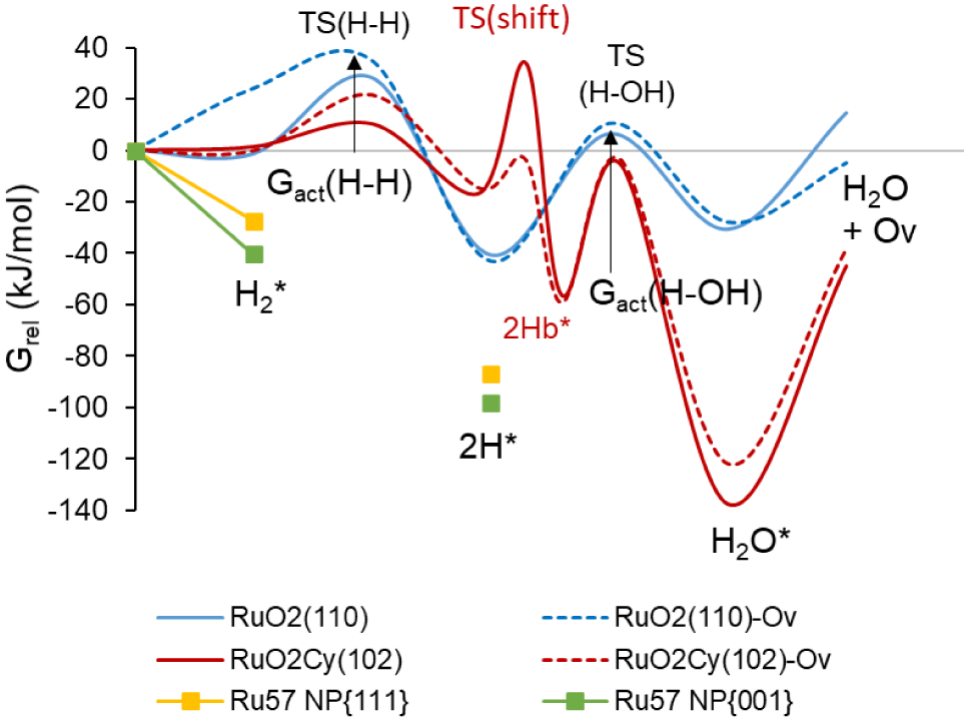
Gibbs free energy profile at 433.15 K for H_2_ reaction with RuO_2_(110) (blue lines) and RuO_2_C_*y*_(102) (red lines) surfaces to form H_2_O and a O vacancy defect, and for H_2_ dissociation on two nanofacets of Ru_57_ nanoparticle (yellow and green plots). The dotted lines correspond to the H_2_ oxidation to H_2_O on RuO_2_(110) and RuO_2_C_*y*_(102) surface models already containing an O vacancy. The arrows indicate the Gibbs activation energies for the H_2_ dissociation and H_2_O formation steps. The calculated Gibbs energy values are given in Table S3.

**Figure 8:**
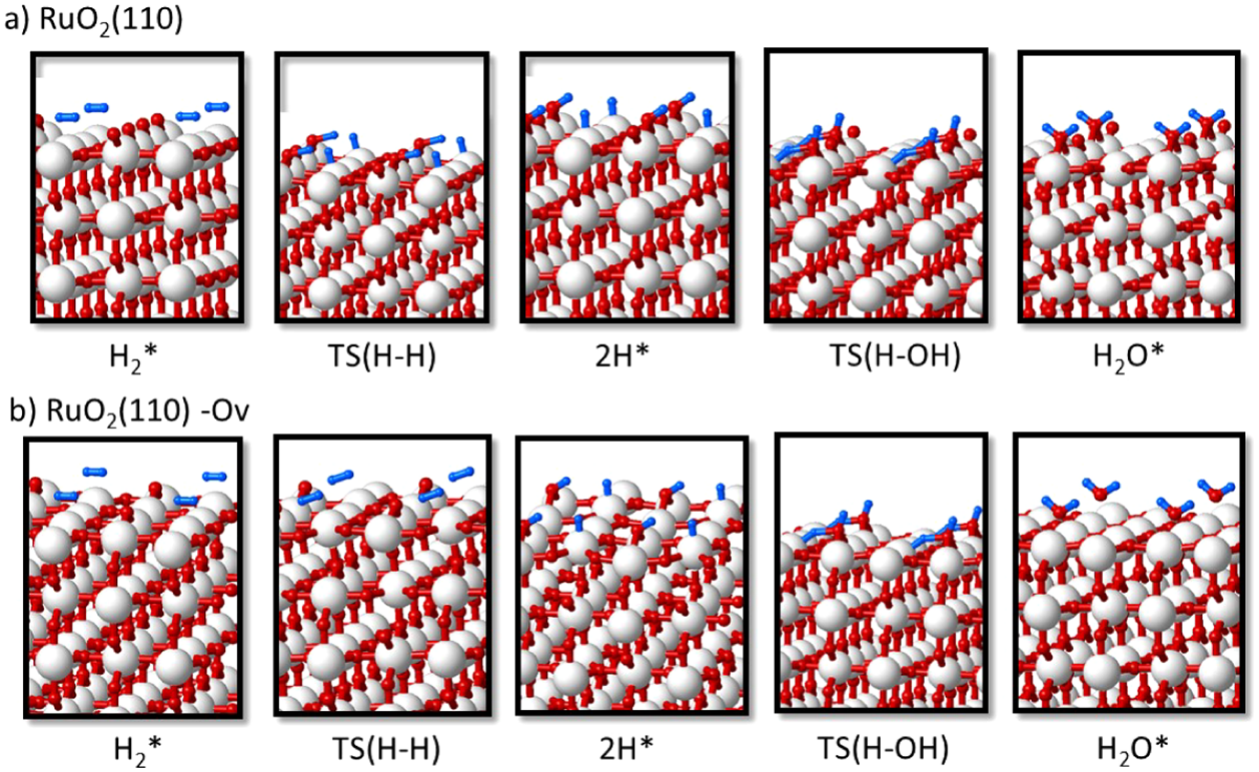
Optimized geometry of all minima and transition state structures involved in the reaction of H_2_ with (a) RuO_2_(110) and (b) RuO_2_(110)-Ov catalyst models to form H_2_O and an O vacancy defect on the surface. Ru, O, and H atoms are depicted as white, red, and blue balls, respectively.

**Figure 9:**
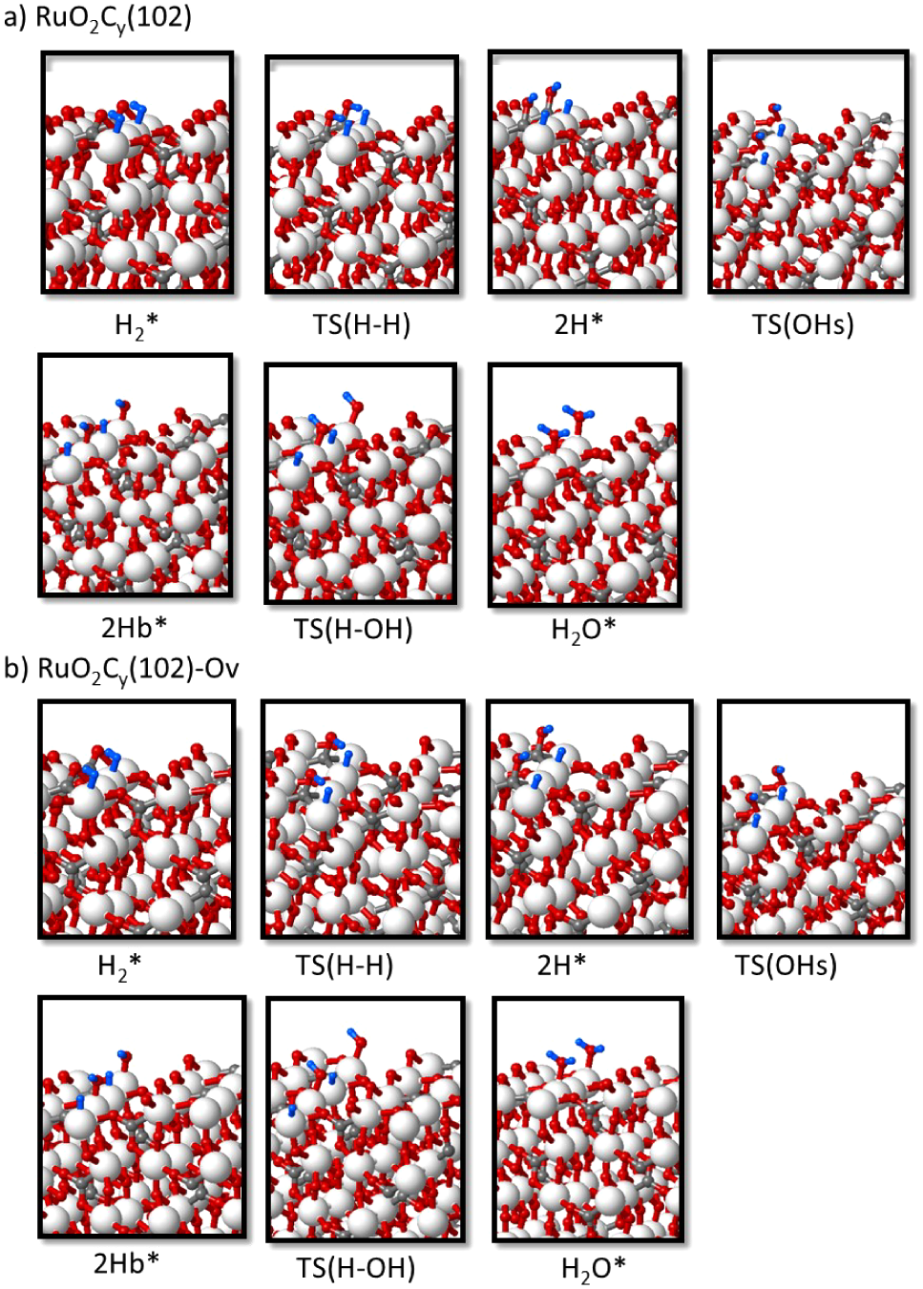
Optimized geometry of all minima and transition state structures involved in the reaction of H_2_ with (a) RuO_2_C_*y*_(102) and (b) RuO_2_C_*y*_(102)-Ov catalyst models to form H_2_O and an O vacancy defect on the surface. Ru, O, C, and H atoms are depicted as white, red, gray, and blue balls, respectively.

The dissociation of H_2_ on the catalyst surface is necessary for the CO_2_ methanation reaction, but it is also the initial step leading to catalyst reduction and deactivation. We investigated the adsorption and dissociation of one H_2_ molecule on different models of the RuO_2_(110) and RuO_2_C_*y*_(102) surfaces, namely the stoichiometric clean surfaces and the partly reduced systems with one oxygen vacancy defect, and the subsequent formation and desorption of H_2_O leaving another O vacancy. The calculated Gibbs energy profiles at 433.15 K are plotted in Figure 7, and the optimized geometries of all adsorbed reactant structures (H_2_*), intermediates (2H^*^), products (H_2_O*), and transition states are depicted in Figures 8 and 9.

In all cases, H_2_ interacts preferentially with a surface Ru atom, forming an adsorption complex with optimized Ru–H distances ranging from 1.8 to 1.9 Å (see Table 2). In the transition state for H_2_ dissociation, TS(H–H), the Ru–H bond strengthens with slightly shorter Ru–H distances, and the other H atom starts interacting with a neighboring O atom, with optimized H–O distances of 1.5 Å for RuO_2_(110) and 1.3 Å for RuO_2_C_*y*_(102). After dissociation forming the 2H* intermediate, all Ru–H bond lengths are 1.6 Å, and all H–O distances are 0.98 Å. The net atomic charges on the two H atoms summarized in Table 3 indicate that the rupture of the H–H bond is heterolytic, with the H atom interacting with Ru getting negatively charged by approximately −0.3 and the other one becoming a proton. The Gibbs activation energy for H_2_ dissociation at 433.15 K is low in all cases, between 9 and 29 kJ/mol (see Table 4) and the process is more exothermic on RuO_2_(110) than on RuO_2_C_*y*_(102) (see Figure 7 and Table S3).

**Table 2:** Optimized Values of the Most Relevant Distances (in Å) in the H_2_ Dissociation Step on the RuO_2_(110) and RuO_2_C_*y*_(102) Surface Models

	H_2_*	TS(H–H)			2H*	
	r(Ru–H)	r(Ru–H)	r(H–H)	r(H–O)	r(Ru–H)	r(H–O)
RuO_2_(110)	1.89	1.84	0.91	1.50	1.63	0.98
RuO_2_C_*y*_(102)	1.80	1.73	1.04	1.31	1.62	0.98
RuO_2_(110)-Ov	1.84	1.81	0.93	1.50	1.62	0.98
RuO_2_C_*y*_(102)-Ov	1.80	1.73	1.06	1.29	1.62	0.98

**Table 3:** Calculated Net Atomic Charges (in e) on the Two H Atoms in the Transition State TS(H–H) and Product (2H*) of the H_2_ Dissociation Step on RuO_2_(110), RuO_2_C_*y*_(102), and Ru_57_ NP Models

	TS(H–H)		2H*	
	qH (Ru)	qH (O)	qH (Ru)	qH (O)
RuO_2_(110)	–0.25	+0.31	–0.30	+1
RuO_*x*_C_*y*_(102)	–0.27	+0.39	–0.26	+1
RuO_2_(110)-Ov	–0.24	+0.29	–0.30	+1
RuO_*x*_C_*y*_(102)-Ov	–0.29	+0.40	–0.25	+1
Ru_57_ NP {101}			–0.32	–0.32
Ru_57_ NP {001}			–0.33	–0.32

**Table 4:** Calculated Adsorption/Desorption Energies of H_2_ and H_2_O, and Activation Free Energies for H_2_ Dissociation and H_2_O Formation at 433.15 K (in kJ/mol) on RuO_2_(110), RuO_2_C_*y*_(102), RuO_2_(110)-Ov and RuO_2_C_*y*_(102)-Ov Surface Models and on Two Nanofacets of Ru_57_ Nanoparticle^a^

	Δ*G*_ads_(H_2_)	G_act_(H–H)	G_act_(H–OH)	Δ*G*_des_(H_2_O)
RuO_2_(110)	–1	29	47	46
RuO_2_C_*y*_(102)	1	9	50	92
RuO_2_(110)-Ov	24	11	53	23
RuO_2_C_*y*_(102)-Ov	0	22	55	84
Ru_57_ NP {101}	–28			
Ru_57_ NP {001}	–40			

a The corresponding energy profiles are plotted in Figure 7.

After heterolytic H_2_ dissociation on RuO_2_(110), the H atom bonded to Ru is transferred through transition state TS(H–OH) to the OH group formed in the first step, yielding adsorbed water (H_2_O* in Figure 8) with a Gibbs activation barrier of 47 kJ/mol, which raises to 53 kJ/mol in the presence of a surface O vacancy (Table 4). The same formation of water requires two steps on the RuO_2_C_*y*_(102) surface. As depicted in Figure 9, the OH group formed in the most favorable pathway for H_2_ dissociation is directly attached to a C atom of the oxycarbonate, and in this situation, water formation is not favored. An additional shift of the OH group to a neighboring Ru atom via transition state TS(OHs) is required, generating a more stable 2Hb* intermediate, which can then accept a H atom through TS(H–OH) and form water adsorbed on the catalyst surface. The Gibbs activation barriers for the OH shift and for the H transfer yielding water on the RuO_2_C_*y*_(102) surface are similar, approximately 50 kJ/mol, and the presence of a surface O vacancy facilitates the shift of the OH group, but not the formation of water with a Gibbs activation barrier of 55 kJ/mol (see Figure 7 and Table 4). Finally, the higher stability of water adsorbed on the RuO_2_C_*y*_(102) surface is remarkable compared to RuO_2_(110). In the former case, the H_2_O molecule is monocoordinated to a surface Ru atom at an optimized Ru–O distance of 2.18 Å (Figure 9), while in the latter case, H_2_O is occupying a bridge position between two surface Ru atoms, with the shortest Ru–O distance being 2.30 Å (Figure 8). These results suggest that while the H_2_ dissociation step is similarly easy on both catalysts, the more difficult water desorption from the RuO_2_C_*y*_(102) surface might also help to prevent its deep reduction.

For comparison, H_2_ dissociation on a Ru_57_ NP was also considered. It was found that the process is clearly exothermic and barrierless (yellow and green plots in Figure 7), since no transition state could be located between reactants and products, and energy continuously decreased as the H_2_ molecule split into two H atoms. The net atomic charges in Table 2, −0.3 e for the two H atoms in all cases, indicate that on metallic Ru^0^ the rupture of the H–H bond is homolytic, and that after dissociation the two negatively charged H atoms tend to occupy hollow positions on the Ru_57_ NP surface (Figure S8).

### 3.6 DFT Study of CO_2_ Interaction with RuO_2_(110), RuO_2_C_*y*_(102), and Ru_57_ NP Catalyst Models

The process competing with RuO_2_ reduction to form metallic Ru^0^ nanoparticles is the formation of the active and stable oxycarbonate phase under reaction conditions, that is, in the presence of CO_2_. To help understand this process, we investigated the adsorption and dissociation of CO_2_ on the same models used in the previous section, namely the stoichiometric and the partly reduced RuO_2_(110) and RuO_2_C_*y*_(102) surfaces, and the metallic Ru_57_ nanoparticle.

**Figure 10:**
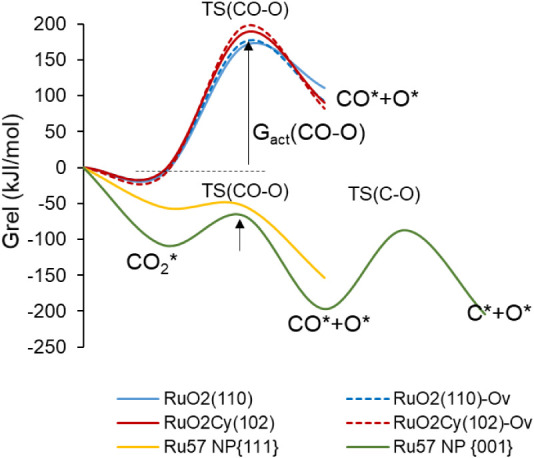
Gibbs free energy profile at 433.15 K for CO_2_ adsorption and dissociation on RuO_2_(110) (blue), RuO_2_C_*y*_(102) (red), and Ru_57_ NP (yellow and green lines) to form CO*+O* and for the subsequent dissociation of CO^*^ into C^*^+O^*^ on the {001} nanofacet of Ru_57_ NP. The arrows indicate the Gibbs activation energy for dissociation of CO_2_ into CO + O. The calculated Gibbs energy values are given in Table S4.

**Figure 11:**
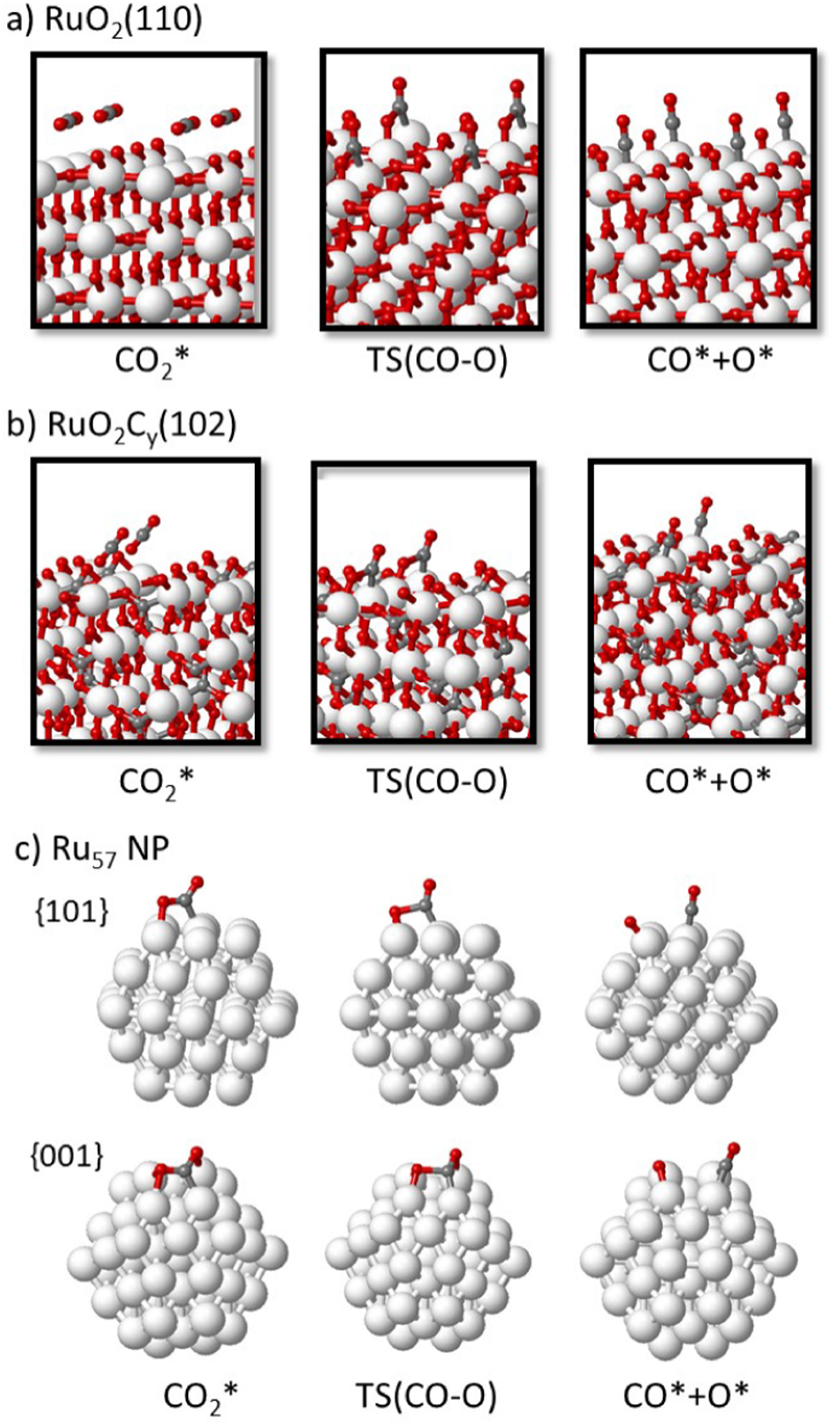
Optimized geometry of reactant, transition state, and product of CO_2_ dissociation into CO+O on (a) RuO_2_(110), (b) RuO_2_C_*y*_(102), and (c) Ru_57_ nanoparticle catalyst models. Ru, O, and C atoms are depicted as white, red, and gray balls, respectively.

The calculated Gibbs energy profiles plotted in Figure 10 and the optimized geometries of the adsorbed CO_2_* reactant depicted in Figures 11 and S9 indicate that the interaction of CO_2_ with the RuO_2_(110) and RuO_2_C_*y*_(102) surfaces is weak and does not lead to molecular activation, neither in the stoichiometric surfaces nor in the partly reduced models. The optimized OCO angle is close to 180° in all systems, and the molecule is always at a distance larger than 3 Å from the closest Ru atom. In the transition state for C–O bond dissociation, TS(CO-O), the C atom of CO_2_ is already attached to a Ru atom at ∼2 Å, the OCO angle is bent, and the C–O bond length increases to 1.8 Å (see Table 5 and Figures 11a,b and S9). After dissociation, the resulting CO molecule remains monocoordinated on top of a Ru atom at an optimized distance of ∼1.9 Å. The Gibbs energy profiles for CO_2_ dissociation on the four RuO_2_(110) and RuO_2_C_*y*_(102) models considered here are equivalent and indicate that the process is kinetically and thermodynamically unfavorable, with Gibbs activation energies higher than 180 kJ/mol (Table 6) and calculated reaction energies larger than 90 kJ/mol (Table S4).

**Table 5:** Optimized Values of the Most Relevant Distances (in Å) in the Dissociation of CO_2_ on RuO_2_(110), RuO_2_C_*y*_(102), and Ru_57_ Models

	CO_2_*		TS(CO-O)			CO*+O*
	r(Ru–C)	a(OCO)	r(Ru–C)	r(C–O)	r(Ru–O)	r(Ru–C)
RuO_2_(110)	3.08	178.9°	2.11	1.77	1.82	1.95
RuO_2_C_*y*_(102)	3.37	189.0°	2.03	1.80	1.85	1.90
RuO_2_(110)-Ov	3.12	179.7°	2.10	1.79	1.82	1.92
RuO_2_C_*y*_(102)-Ov	3.36	178.7°	2.03	1.80	1.84	1.90
Ru_57_ NP {101}	2.08	127.2°	2.03	1.72	2.04	1.87
Ru_57_ NP {001}	2.03	119.4°	1.99	1.85	1.99	2.04

**Table 6:** Calculated Adsorption and Activation Gibbs Energies for CO_2_ Dissociation at 433.15 K (in kJ/mol) on RuO_2_(110), RuO_2_C_*y*_(102), RuO_2_(110)-Ov, and RuO_2_C_*y*_(102)-Ov Surface Models and on Two Nanofacets of Ru_57_ Nanoparticle^a^

	Δ*G*_ads_(CO_2_)	*G*_act_(CO–O)	*G*_act_(C–O)
RuO_2_(110)	–8	178	
RuO_2_C_*y*_(102)	–5	180	
RuO_2_(110)-Ov	–4	191	
RuO_2_C_*y*_(102)-Ov	–10	207	
Ru_57_ NP {101}	–56	2	
Ru_57_ NP {001}	–108	41	110

a The corresponding energy profiles are plotted in Figure 10.

**Figure 12:**
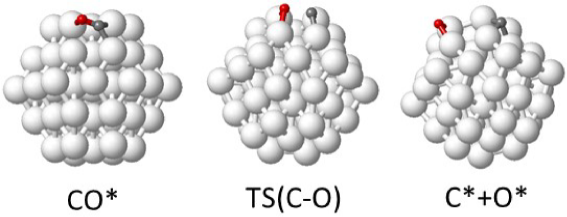
Optimized geometry of reactant, transition state, and product of CO dissociation into C+O on a Ru_57_ nanoparticle. Ru, O, and C atoms are depicted as white, red, and gray balls, respectively.

In contrast, CO_2_ chemisorption on the metallic Ru_57_ nanoparticle is exothermic and leads to noticeable molecular bending (see Figures 10, 11c, and Table 3). Once activated, the rupture of the C–O bond via TS (CO-O) is easy and thermodynamically favored. The optimized structures depicted in Figure 11c explain the different Gibbs energy profiles obtained for the reaction taking place at the {111} and {001} facets of the Ru_57_ nanoparticle. In the former, CO_2_ adsorbs forming two new bonds (Ru–O and Ru–C) with the metal surface, while in the latter, an additional bond is formed between the second O atom of CO_2_ and another Ru atom of the active site, which results in a more stable adsorption complex. The higher stability of CO_2_ adsorbed on the {001} plane leads to a higher Gibbs activation energy for C–O bond dissociation, 41 kJ/mol versus only 2 kJ/mol on the {111} facet (see Figure 10 and Table 6). After dissociation on the {001} facet, the resulting CO molecule remains with the C atom bicoordinated to two Ru atoms, in an orientation that facilitates the interaction of O with another Ru atom and its subsequent dissociation into C + O (see Figures 10 and 12). The Gibbs activation energy for this step is 110 kJ/mol, and the process is slightly exothermic.

Comparison of the lowest Gibbs activation energy for the dissociation of CO into C+O, 110 kJ/mol on the metallic Ru_57_ NP, with that previously obtained for RuO_2_(110) reduction by reaction with H_2_, around 50 kJ/mol, confirms the proposal that RuO_2_ reduction into metallic Ru^0^ is a fast process under the reaction conditions. An adequate balance between Ru^0^ and RuO_2_C_*y*_ was reported in our previous work^9^ as necessary for catalyst activity and selectivity; Ru^0^ alone is inactive under our reaction conditions, and the presence of RuO_2_C_*y*_ is key for catalyst activity.

The data presented here suggest that while the H_2_ dissociation step is possible on the three phases considered (Ru^0^, RuO_2_ and RuO_2_C_*y*_), the dissociation of CO_2_ into CO+O requires the presence of some small metallic Ru^0^ nanoparticles. Moreover, metallic Ru^0^ is necessary for the dissociation of CO into C+O, where the generated C atoms act in part as a reservoir stabilizing the RuO_2_C_*y*_ phase. This study describes the role of the different phases present in the catalyst, while the full reaction path for CO_2_ methanation on this material will be investigated in further work.

## 4 Conclusions

Recently, we reported a new ruthenium-based catalyst for low-temperature CO_2_ methanation, labeled RuO_*x*_C_*y*_@C, which is composed of three phases: RuO_2_C_*y*_, RuO_2_, and Ru^0^, each of them playing a role in the activity and long-term stability of the material. In our previous work, we demonstrated RuO_2_C_*y*_ as the active phase in the reaction, which can also be generated in situ under reaction conditions starting from RuO_2_ as the precatalyst. However, catalyst stability is influenced by the kinetics of two processes: the reduction of RuO_2_ to inactive metallic Ru^0^ and the in situ formation of the active RuO_2_C_*y*_ phase under reaction conditions via C diffusion into the RuO_2_ lattice. Herein, we have investigated both processes using a combination of experimental and computational techniques,

The catalytic studies in the hydrogenation of CO_2_ to methane show a high initial activity of RuO_2_ that drops quickly under reaction conditions, in contrast to a stable catalytic performance of the RuO_*x*_C_*y*_@C material for several days. XRD and XPS characterization of the two samples at different reaction times shows that in the initial stages of the reaction, RuO_2_ is partly converted into RuO_2_C_*y*_, but after some hours, the sample is almost completely transformed into inactive Ru^0^. In contrast, only a small amount of metallic Ru^0^ is formed in the RuO_*x*_C_*y*_@C sample, confirming that RuO_2_C_*y*_ is the active phase of the catalyst. A second experiment done in the presence of only H_2_ confirms the fast reduction of RuO_2_ to metallic Ru^0^ and demonstrates that the presence of interstitial C in the ruthenium oxycarbonate phase slows down this reduction process. This conclusion is supported by DFT calculations of bulk reducibility. The energy necessary to form oxygen vacancies in RuO_2_ decreases continuously as the material gets reduced, while in RuO_2_C_*y*_ the electrons left in the solid after O removal are accepted by the interstitial C atoms, stabilizing a cationic oxidation state of Ru.

Periodic DFT calculations of the surface reactivity indicate that both RuO_2_(110) and RuO_2_C_*y*_(102) dissociate H_2_ heterolytically and form water with similar Gibbs activation energies. However, water desorption from RuO_2_C_*y*_(102) is somewhat hindered, which might help prevent its deep reduction. On the other hand, CO_2_ dissociation into CO+O is highly endothermic on both RuO_2_(110) and RuO_2_C_*y*_(102) surfaces, but energetically accessible on metallic Ru^0^ nanoparticles. Comparison of all calculated Gibbs activation energies supports the proposal of a fast reduction of RuO_2_ with H_2_ to form Ru^0^ nanoparticles, and a slower rupture of CO_2_ on these Ru^0^ nanoparticles to generate the C atoms that diffuse into RuO_2_ to form the active and stable RuO_2_C_*y*_ phase.

## Supporting Information Available

Deactivation rates of catalysts; Additional characterization of fresh and spent samples; Detailed description of the procedure and methods used to determine the kinetics of the ruthenium oxide reduction; Optimized structures involved in H_2_ dissociation on Ru_57_ and in CO_2_ reaction on RuO_2_(110)-Ov, and RuO_2_C_*y*_(102)-Ov models; Calculated relative Gibbs free energies and adsorption, desorption, and activation barriers (DOCX)
