# Supplementary material for: Unlocking the Role of C Doping in a RuO2 Matrix in CO2 Methanation from a Combined Theoretical and Experimental Approach
Source: J Phys Chem C Nanomater Interfaces. 2025 Mar 12;129(11):5449–60. doi: 10.1021/acs.jpcc.5c01277 (PMC11931542; doi:10.1021/acs.jpcc.5c01277)
Supplement: Supplementary file 1 — jp5c01277_si_001.docx [file jp5c01277_si_001.docx]

Unlocking the Role of C Doping in a RuO_2_ Matrix in the CO_2_ Methanation from a Combined Theoretical and Experimental Approach

*Alvaro Royo de Larios, Carmen Tébar-Soler, Daviel Gómez, Patricia Concepción,* Mercedes Boronat,* Avelino Corma**

Instituto de Tecnología Química, Universitat Politècnica de València – Consejo Superior de Investigaciones Científicas, Avenida de los Naranjos s/n, 46022 València, Spain

**1. Catalytic data**

**Deactivation rate of RuO_2_ and RuO_x_C_y_@C catalyst at 20bar and 180ºC.**

The deactivation constant (K_D_) has been calculated for the catalytic data displayed in figure 2 of the manuscript for both RuO₂ and RuO_x_C_y_@C catalysts, corresponding to reaction conditions of 180 ºC, 20bar and 24000h^-1^. To quantitatively evaluate deactivation, the activity curves were analyzed using a second-order kinetic deactivation model (Equations 1-3).

$$\begin{aligned} a=\frac{{STY}_{t=t}}{{STY}_{t=max}}\#\left( 1 \right) \end{aligned}$$

$$\begin{aligned} {-\frac{da}{dt}=K}_{D}a^{2}\#\left( 2 \right) \end{aligned}$$

$$\begin{aligned} \frac{1}{a}=\frac{1}{a_{0}}+K_{D}t\#\left( 3 \right) \end{aligned}$$

As can be seen in Figure S1, the deactivation constant is higher (i.e., 0.0005) for RuO₂ compared to the RuO_x_C_y_@C (i.e., 0.00003). This is due to the greater reducibility of RuO₂, which makes it more susceptible to deactivation over time. In contrast, the RuO_x_C_y_@C catalyst exhibits higher stability, likely because its oxycarbonate structure provides better resistance to reduction under the reaction conditions. This difference in deactivation behaviour highlights the impact of catalyst composition on long-term performance.


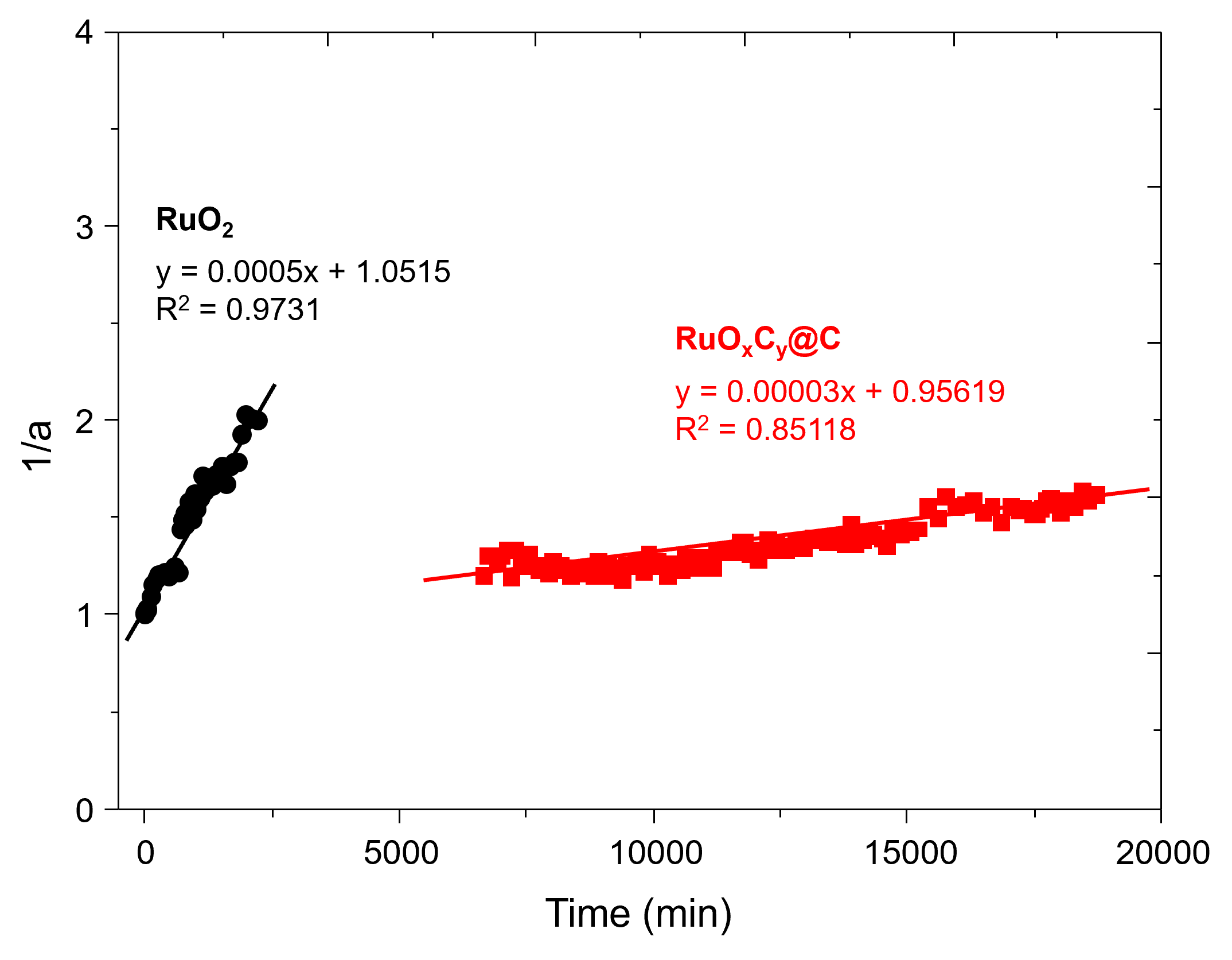


**Figure S1**. Deactivation constants values for RuO₂ (in black) and RuO_x_C_y_@C (in red) fitted to a second-order deactivation model. Note that the values of STY corresponding to the inductions period were not taken into account.

**Catalytic data at 160 ºC and 10 bar.** The catalytic data at 160 ºC, 10 bar and 60000h^-1^ obtained in a fixed bed reactor are given in Figure S2.

**
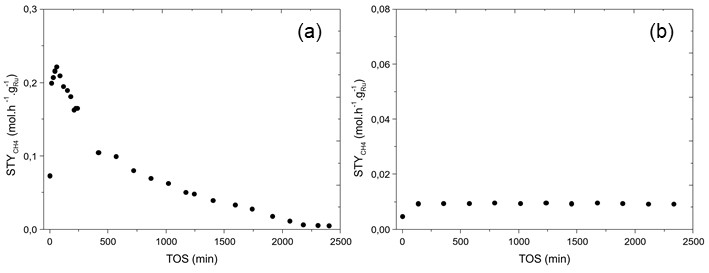
**

**Figure S2.** Space time yield of methane (STY) in mol.h^-1^.g_Ru_^-1^ over (a) RuO_2_ and (b) RuO*_x_*C*_y_*@C samples. Reaction conditions: 10 bar, 160 °C, CO_2_:H_2_ 1:3, and 60000 h^-1^.

**2. Catalyst characterization: fresh and used samples**

**X-ray photoelectron spectroscopy (XPS) of fresh and used RuO_2_ and RuO_x_C_y_@C samples.** The XPS spectra obtained on fresh and used samples are given in Fig. S3 and summarized in Table 1 (main text).

**Figure S3.** XPS of the C 1s and Ru 3d core levels on RuO_x_C_y_@C sample: (a) fresh, (b) after 1 min and (c) after 30 min exposed to reaction conditions (10 bar, 160 ºC and 1:3 CO_2_:H_2_), and RuO_2_ sample: (d) fresh and (e) after 30 min exposed to reaction conditions. Colour code for components: ruthenium oxy‑carbonate (labelled as RuO_x_C_y_) in red, metallic ruthenium (labelled as Ru 0) in green, oxidize ruthenium (labelled as Ru IV and Ru VI) in dark blue and cyan, respectively, and carbon in grey.

**Temperature programmed reduction in H_2_ (TPR-H_2_) of fresh RuO_2_ and RuO_x_C_y_@C samples.** TPR‐H_2_ analyses of Figure S4 were performed on a Micromeritics Autochem 2910 instrument. 50 mg of sample were initially cleaned with 30 mL∙min^‐1^ of Ar at room temperature for 30 min. Then, a feed of 10 vol % of H_2_ in Ar (50 mL∙min^‐1^) was passed through the solid while the temperature was increased up to 600 °C (10 ºC·min^-1^).


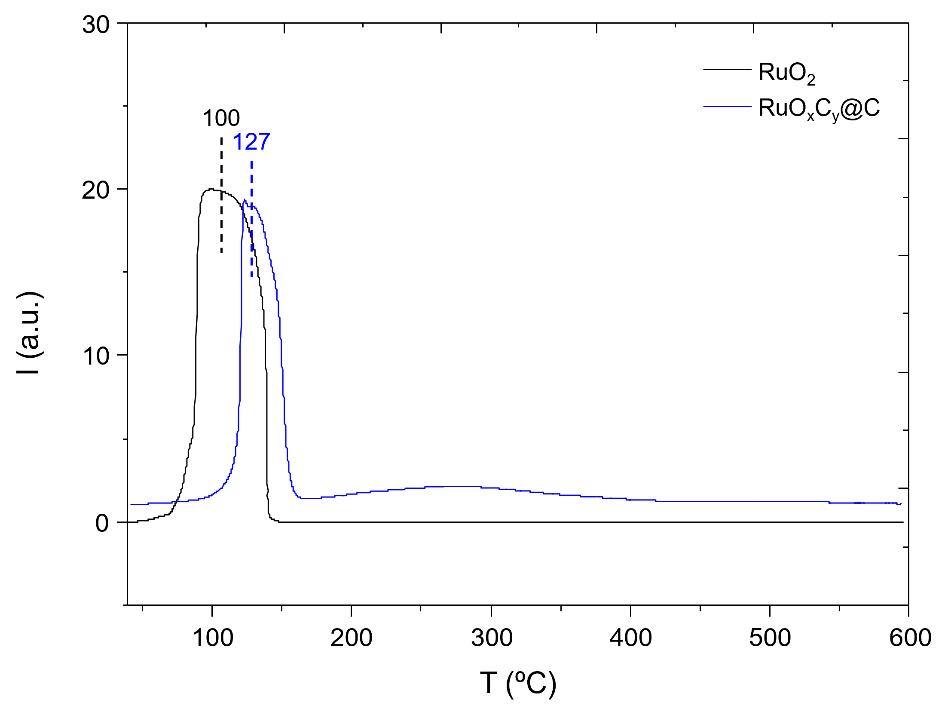


**Figure S4.** TPR‐H_2_ of RuO_2_ (in black) and fresh RuO_x_C_y_@C sample (in blue). The maxima temperature is indicated in the graph with a dotted line.

**3. Ruthenium oxide reduction**

The reduction reaction of ruthenium oxide upon exposure to hydrogen forms water as the main product according to the overall reaction:

$$RuO_{2}+2H_{2}\to Ru+2H_{2}O (1)$$

The reduction mechanism has been previously studied by another group (1), but in this work a specific mechanism is not considered. Until now, several methods have been proposed to calculate the kinetic parameters of the reduction process of metal oxides from TPR measurements such as the Stationary point method (2), Kissinger method (3), Friedman method (4), Flynn-Wall-Ozawa method (5, 6) and Kissinger-Akahira-Sunose method (7). However, all of them are based on the general expression of the reduction rate, or in other words, the rate of water formation (r_H2O_=r_Red_):

$$r_{H_{2}O}=r_{Red}=Ae^{-\frac{Eapp}{RT}}\left[ H_{2} \right]^{\delta}\left[ S \right]^{\beta} (2)$$

where [S] represents the concentration of RuO_2_, A is the pre-exponential factor, δ and β represent the orders of H_2_ and RuO_2_ respectively, R is the gas constant and E_app_ is the apparent activation energy.

The degree of reduction of RuO_2_ into Ru (α) can be calculated as:

$$\alpha=\frac{{S_{t}-S}_{0}}{{S_{f}-S}_{0}} (3)$$

where S_t_, S_0_ and S_f_ represent the signal of H_2_O (*m/z* 18) at the temporal, initial and final instant. Furthermore, the reduction rate (dα/dt) can be written as:

$$r_{Red}=\frac{d\alpha}{dt}=\frac{\alpha_{f}-\alpha_{i}}{t_{f}-t_{i}} (4)$$

$$\frac{d\alpha}{dt}=\frac{dT}{dt}\frac{d\alpha}{dT} (5)$$

where *d*T/*d*t is the heating rate of TPR. The apparent activation energy of RuO_2_ reduction under linear heating can be calculated without knowing the specific reduction mechanism according to the expressions in Table S1.


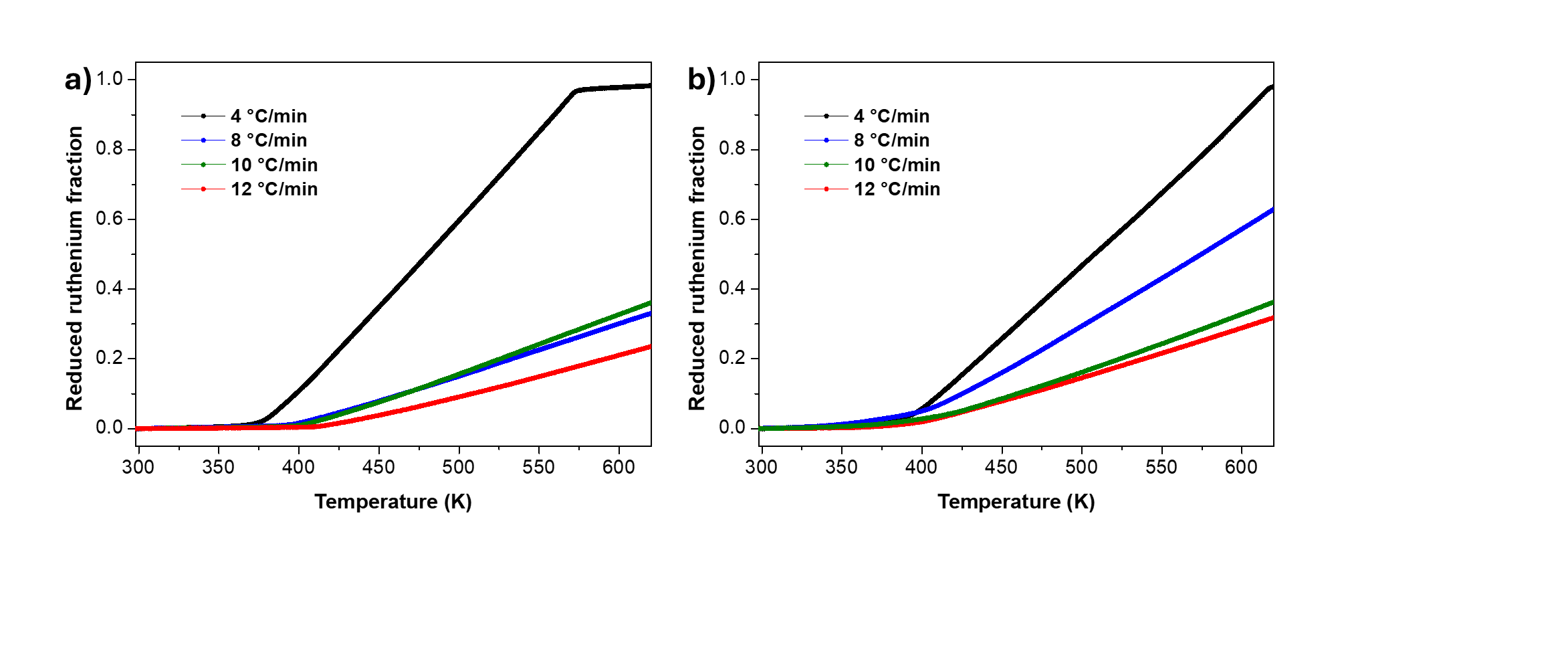


**Figure S5.** Degree of conversion curve as a function of temperature for the reduction of ruthenium oxide under hydrogen at different heating rate (4, 8, 10 and 12 °C/min) in samples a) RuO_2_ and b) RuO_x_C_y_@C. Experimental conditions: 50 mg of sample and 15 mL/min of 33%H_2_/He mixture.

**Table S1.** Calculation methods used to determine the estimated value of the apparent activation energy of the ruthenium oxide reduction process.

| **Method** | **Logarithmic form of expresion** | **Observations** |
| --- | --- | --- |
| ***Stationary point*** | $\ln\left[ \beta\left( \frac{d\alpha}{dT_{max}} \right) \right]=-\frac{E_{app}}{RT_{max}}+\ln\left[ Af\left( \alpha_{max} \right) \right]$ | Local maximum (Temperature at the maximal velocity) |
| $\beta=\frac{dT}{dt}$ is the heating rate; $T_{max}$ is the temperatura at the maximal velocity of process $\left( \frac{d\alpha}{dt} \right)$ or at $\frac{d^{2}\alpha}{dt^{2}}=0$; $f\left( \alpha_{max} \right)$ is a function of the reaction mechanism | | |
| ***Kissinger*** | $\ln\left[ \frac{\beta}{T_{max}^{2}} \right]=-\frac{E_{app}}{RT_{max}}+\ln\left[ \frac{AR}{E_{app}} \right]$ | Temperature at maximum reaction rate in constant heating rate |
| $\beta=\frac{dT}{dt}$ is the heating rate; $T_{max}$ is the temperatura at the maximal velocity of process $\left( \frac{d\alpha}{dt} \right)$ or at $\frac{d^{2}\alpha}{dt^{2}}=0$ | | |
| ***Friedman*** | $\ln\left[ \beta\left( \frac{d\alpha}{dT_{\alpha_{i}}} \right) \right]=-\frac{{E_{app}}_{i}}{RT_{\alpha_{i}}}+\ln\left[ Af\left( \alpha_{i} \right) \right]$ | Isoconversional differential method |
| $\beta=\frac{dT}{dt}$ is the heating rate; $T_{\alpha_{i}}$ is the temperatura at a specific value of conversion $(\alpha_{i})$; ${E_{app}}_{i}$ is expresed as a function of $\alpha_{i}$ | | |
| ***Flynn-Wall-Ozawa*** | $\ln\left[ \beta\right]=-1.052\frac{E_{app}}{RT_{\alpha_{i}}}-5.331+\ln\left[ \frac{AE_{app}}{Rg(\alpha)} \right]$ | Isoconversional integral method |
| $\beta=\frac{dT}{dt}$ is the heating rate; ; $T_{\alpha_{i}}$ is the temperatura at a specific value of conversion $(\alpha_{i})$; $g\left( \alpha_{i} \right)$ is the algebraic expression for integral form of the reaction model | | |
| ***Kissinger-Akahira-Sunose*** | $\ln\left[ \frac{\beta}{T_{\alpha_{i}}^{2}} \right]=-\frac{E_{app}}{RT_{\alpha_{i}}}+\ln\left[ \frac{AR}{E_{app}g(\alpha)} \right]$ | Isoconversional integral method |
| $\beta=\frac{dT}{dt}$ is the heating rate; ; $T_{\alpha_{i}}$ is the temperatura at a specific value of conversion $(\alpha_{i})$; $g\left( \alpha_{i} \right)$ is the algebraic expression for integral form of the reaction model | | |

Experimentally, TPR curves at linear heating rates of 4, 8, 10 and 12 °C/min of 50 mg of RuO_2_ and RuO_x_C_y_@C samples were used to estimate the apparent activation energy parameter from the Arrhenius plot of each of the above-mentioned methods (Figures S5 and S6). The results are summarized in Table S2.


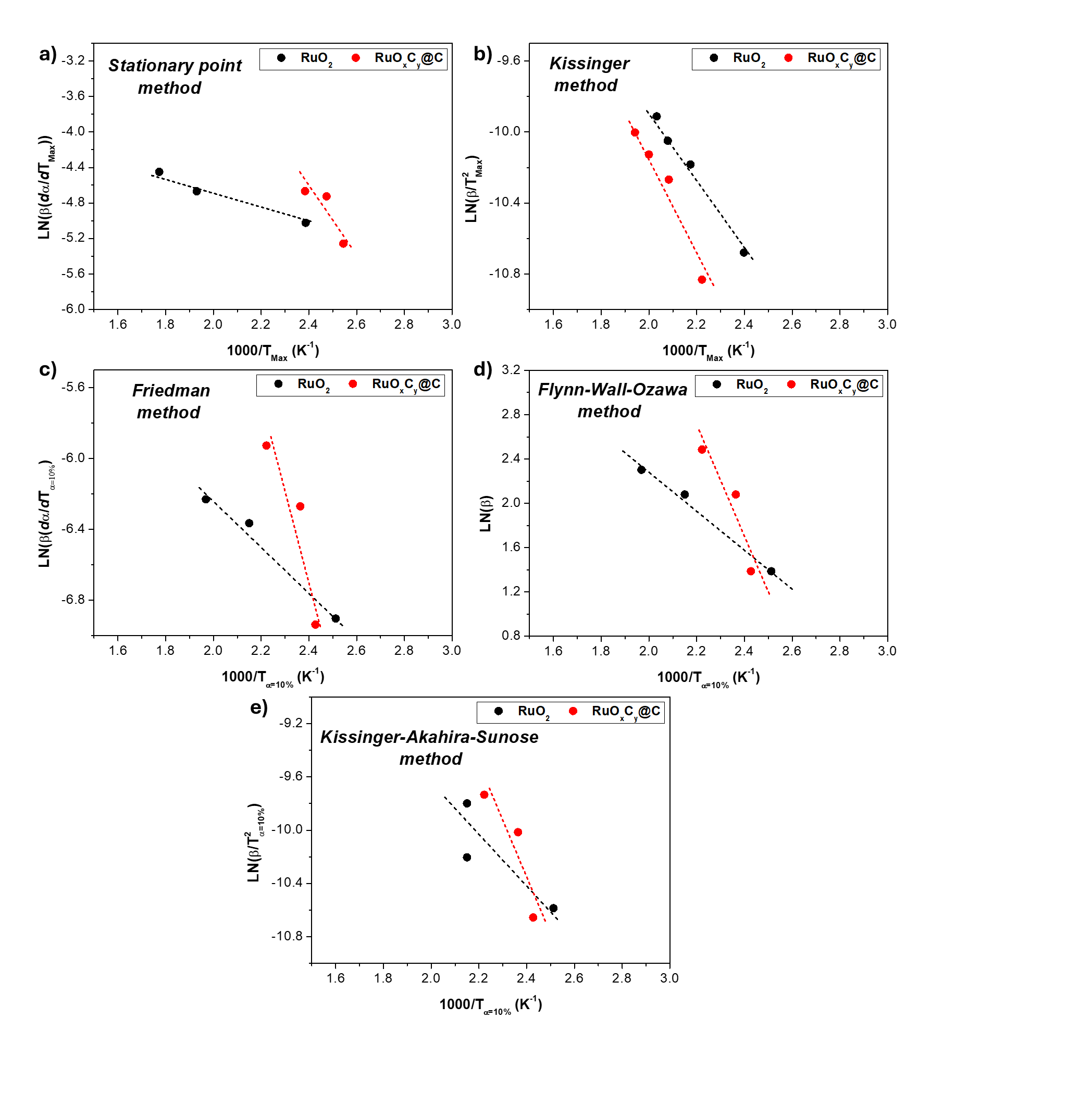


**Figure S6.** a) Stationary point plot, b) Kissinger plot, c) Friedman plot, d) Flynn-Wall-Ozawa plot and e) Kissinger-Akahira-Sunose plot for the reduction of ruthenium oxide under hydrogen in RuO_2_ and RuO_x_C_y_@C samples. Experimental conditions: heating rate (4, 8, 10 and 12 °C/min), 50 mg of sample and 15 mL/min of 33%H_2_/He mixture.

**Table S2.** Apparent activation energy values ​​of ruthenium oxide reduction determined from different methods.

| **Sample** | **Apparent activation energy of reduction (kJ/mol)** | | | | |
| --- | --- | --- | --- | --- | --- |
|  | ***Stationary point*** | ***Kissinger*** | ***Friedman***  $(\alpha_{i}=10\%)$ | ***Flynn-Wall-Ozawa***  $(\alpha_{i}=10\%)$ | ***Kissinger-Akahira-Sunose***  $(\alpha_{i}=10\%)$ |
| *RuO_2_* | 7.5 | 17.1 | 10.6 | 13.6 | 13.5 |
| *RuO_x_C_y_@C* | 29.6 | 24.5 | 37.5 | 39.0 | 33.9 |

**4. Additional DFT results**


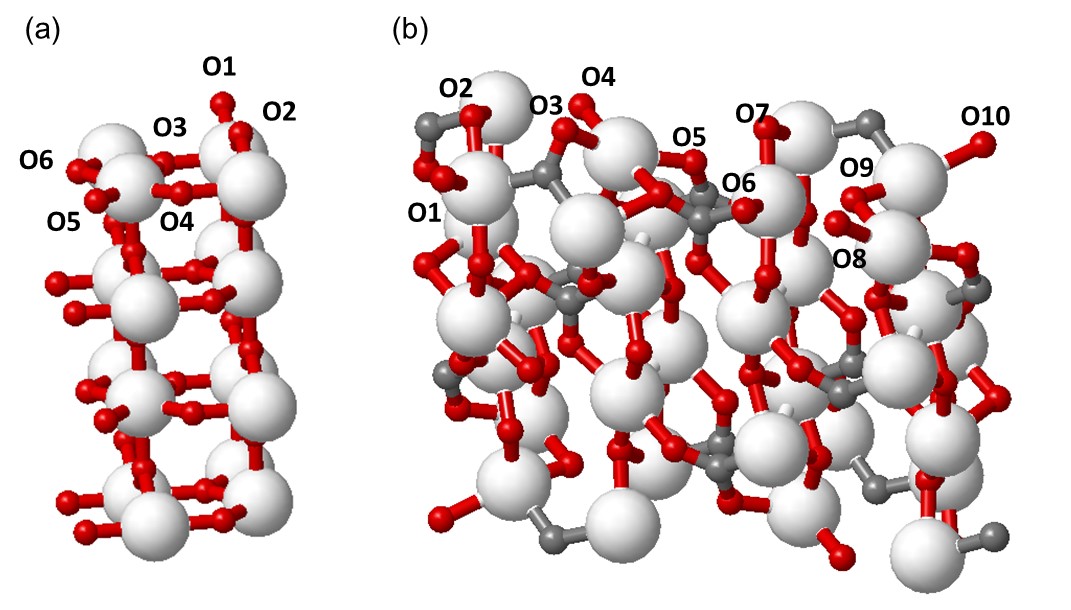


**Figure S7.** Numbering of the oxygen atoms at the surface of a) RuO_2_(110) and b) RuO_2_C_y_ (102) models.


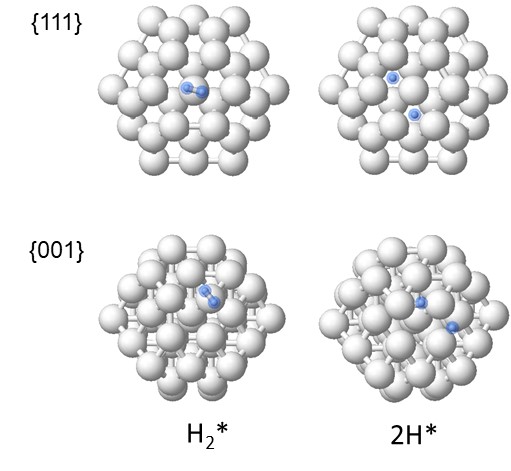


**Figure S8.** Optimized geometry of reactant (H_2_*) and product (2H*) of the H_2_ dissociation process on two different facets of a Ru_57_ nanoparticle model. Ru and H atoms are depicted as white and blue balls, respectively.


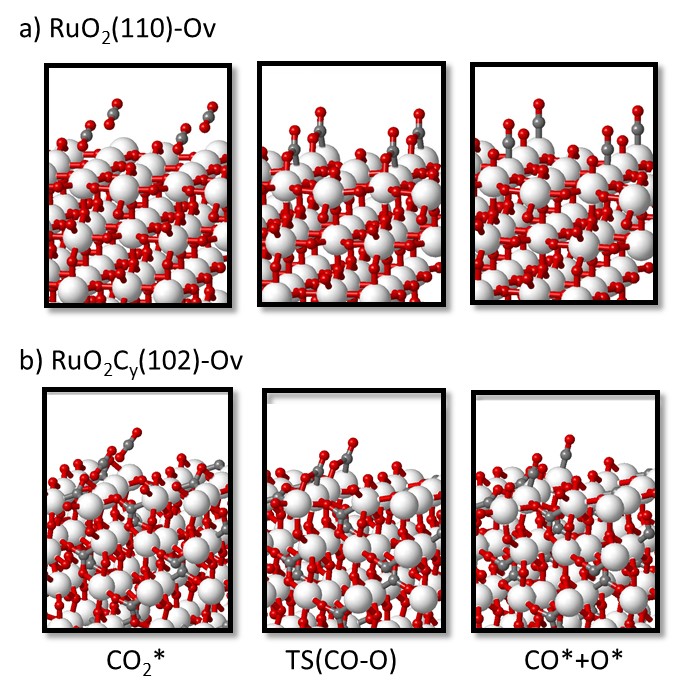


**Figure S9.** Optimized geometry of reactant, transition state and product of CO_2_ dissociation into CO+O on a) RuO_2_(110)-Ov, and b) RuO_2_C_y_(102)-Ov catalyst models. Ru, O and C atoms are depicted as white, red and grey balls.

**Table S3.** Calculated relative Gibbs free energies at 433.15 K (in kJ/mol) of all structures involved in the reaction of H_2_ with RuO_2_(110), RuO_2_C_y_(102), RuO_2_(110)-Ov and RuO_2_C_y_(102)-Ov surface models to form H_2_O and a O vacancy defect, and in H_2_ dissociation on two nanofacets of Ru_57_ nanoparticle. The corresponding Gibbs energy profiles are depicted in Figure 7. The zero energy is in all cases the sum of the energy of the catalyst model and H_2_.

|  | H_2_* | TS(H-H) | 2H* | TS(OHs) | 2Hb* | TS(H-OH) | H_2_O* | H_2_O+Ov |
| --- | --- | --- | --- | --- | --- | --- | --- | --- |
| RuO_2_(110) | -1 | 28 | -41 |  |  | 6 | -31 | 15 |
| RuO_2_C_y_(102) | 1 | 10 | -17 | 34 | -56 | -6 | -137 | -45 |
| RuO_2_(110)-Ov | 24 | 35 | -43 |  |  | 10 | -28 | -5 |
| RuO_2_C_y_(102)-Ov | 0 | 22 | -15 | -4 | -59 | -4 | -122 | -38 |
| Ru_57_ NP {101} | -28 |  | -87 |  |  |  |  |  |
| Ru_57_ NP {001} | -40 |  | -98 |  |  |  |  |  |

**Table S4.** Calculated relative Gibbs free energies at 433.15 K (in kJ/mol) of all structures involved in the dissociation of CO_2_ on RuO_2_(110), RuO_2_C_y_(102), RuO_2_(110)-Ov and RuO_2_C_y_(102)-Ov surface models and on two nanofacets of Ru_57_ nanoparticle. The corresponding Gibbs energy profiles are depicted in Figure 10. The zero energy is in all cases the sum of the energy of the catalyst model and CO_2_.

|  | CO_2_* | TS(CO-O) | CO*+O* | TS(C-O) | C*+O* |
| --- | --- | --- | --- | --- | --- |
| RuO_2_(110) | -8 | 170 | 111 |  |  |
| RuO_2_C_y_(102) | -5 | 175 | 92 |  |  |
| RuO_2_(110)-Ov | -4 | 187 | 90 |  |  |
| RuO_2_C_y_(102)-Ov | -10 | 197 | 83 |  |  |
| Ru_57_ NP {101} | -56 | -54 | -153 |  |  |
| Ru_57_ NP {001} | -108 | -67 | -197 | -87 | -204 |

**Table S4.** Calculated adsorption/desorption energies of H_2_ (ΔG_ads_(H_2_)), H_2_O (ΔG_des_(H_2_O)), and CO_2_ (ΔG_ads_(CO_2_)), and activation free energies for H_2_ dissociation (G_a_(H-H)), H_2_O formation (G_a_(H-OH)) and CO_2_ dissociation (G_a_(CO-O)) at 433.15 K (in kJ/mol) on RuO_2_(110), RuO_2_C_y_(102), RuO_2_(110)-Ov and RuO_2_C_y_(102)-Ov surface models and on two nanofacets of Ru_57_ nanoparticle. The corresponding energy profiles are plotted in Figures 7 and 10.

|  | ΔG_ads_(H_2_) | G_a_(H-H) | G_a_(H-OH) | ΔG_des_(H_2_O) | ΔG_ads_(CO_2_) | G_a_(CO-O) |
| --- | --- | --- | --- | --- | --- | --- |
| RuO_2_(110) | -1 | 29 | 47 | 46 | -8 | 178 |
| RuO_2_C_y_(102) | 1 | 9 | 50 | 92 | -5 | 180 |
| RuO_2_(110)-Ov | 24 | 11 | 53 | 23 | -4 | 191 |
| RuO_2_C_y_(102)-Ov | 0 | 22 | 55 | 84 | -10 | 207 |
| Ru_57_ NP {101} | -28 |  |  |  | -56 | 2 |
| Ru_57_ NP {001} | -40 |  |  |  | -108 | 41 |

REFERENCES

(1) M Knapp, J. Phys. Chem. C 2012, 116, 26822−26828

(2) I Klarić. Journal of Thermal Analysis and Calorimetry, (1995), 45(6), 1373-1380),

(3) H. E.Kissinger, Analytical Chemistry, 1957, 29, 1702–1706),

(4) H.L. Friedman. Journal of Polymer Science Part C, 1963, 6, 183–195),

(5) J. H. F. Flynn, L. A. Wall, Journal of Research of the National Bureau of Standards—A. Physics and Chemistry, 1966, 70, 487–523.

(6) T Ozawa. Bulletin of Chemical Society of Japan, 1965, 38, 1881–1886.

(7) T. Akahira, T. T. Sunose. Research Report Chiba Institute and Technology, 1971, 16, 22–31.
